# Supplementary material for: Cancer-Associated Gain-of-Function Mutations Activate a SWI/SNF-Family Regulatory Hub
Source: Mol Cell. 2020 Nov 19;80(4):712–725.e5. doi: 10.1016/j.molcel.2020.09.024 (PMC7853424; doi:10.1016/j.molcel.2020.09.024)
Supplement: Document S1. Figures S1–S6 and Tables S1 and S2 [file mmc1.pdf]

Molecular Cell, Volume *80*

Supplemental Information

Cancer-Associated Gain-of-Function Mutations Activate a SWI/SNF-Family Regulatory Hub

Cedric R. Clapier, Naveen Verma, Timothy J. Parnell, and Bradley R. Cairns

SUPPLEMENTAL INFORMATION

Cancer-Associated Gain-of-Function Mutations Activate a SWI/SNF-Family Regulatory Hub

Cedric R. Clapier,[1,2,3,*] Naveen Verma,[1,2,3] Timothy J. Parnell,[2] and Bradley R. Cairns[1,2,*]

[1]Department of Oncological Sciences and Howard Hughes Medical Institute, University of Utah School of Medicine, Salt Lake City, UT 84112, USA
[2]Huntsman Cancer Institute, University of Utah School of Medicine, Salt Lake City, UT 84112, USA
[3]These authors contributed equally
[*]Correspondence: brad.cairns@hci.utah.edu or cedric.clapier@hci.utah.edu
Lead Contact: brad.cairns@hci.utah.edu

Figure S1, related to Figures 2, 3 and 4.
Schematic of DNA Translocation Assays.

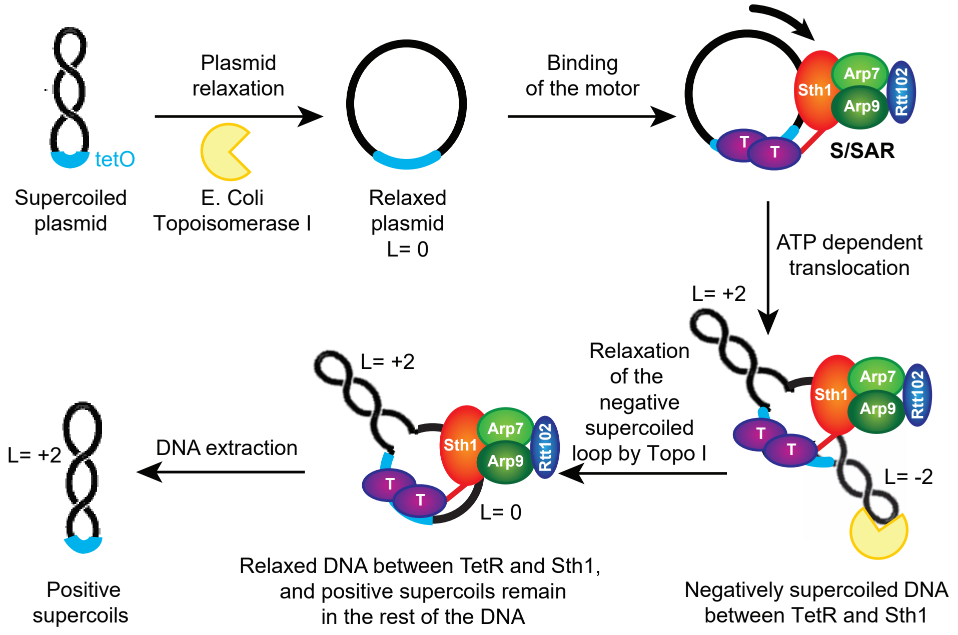

Principle of the tethered translocation assay. Supercoiled topoisomers are proportional to DNA translocation by a tethered Sth1. Sth1 is fused to the Tet repressor (T-Sth1), which forms a heterodimer with an unfused Tet repressor (T). A Tet-Sth1 complex, with (**SAR**) or without (**S**) the Arp7-Arp9-Rtt102 module, anchors/tethers to a single *tetO* operator present on a plasmid DNA, which has previously been relaxed by *E. coli* Topoisomerase I. The ATPase domain of Sth1 engages the DNA and performs ATP-dependent DNA translocation. Translocation of Sth1 along the DNA backbone generates negative supercoils between the enzyme and the *tetO* anchor, and positive supercoils in front of the enzyme. The negative supercoils are specifically relaxed by *E. coli* Topoisomerase I, resulting in the accumulation of positive supercoils that can be visualized after deproteinization on an agarose gel.

Figure S2, related to Figures 2 and 6.
Alanine Scanning, and Addition of the ARP Module.

A

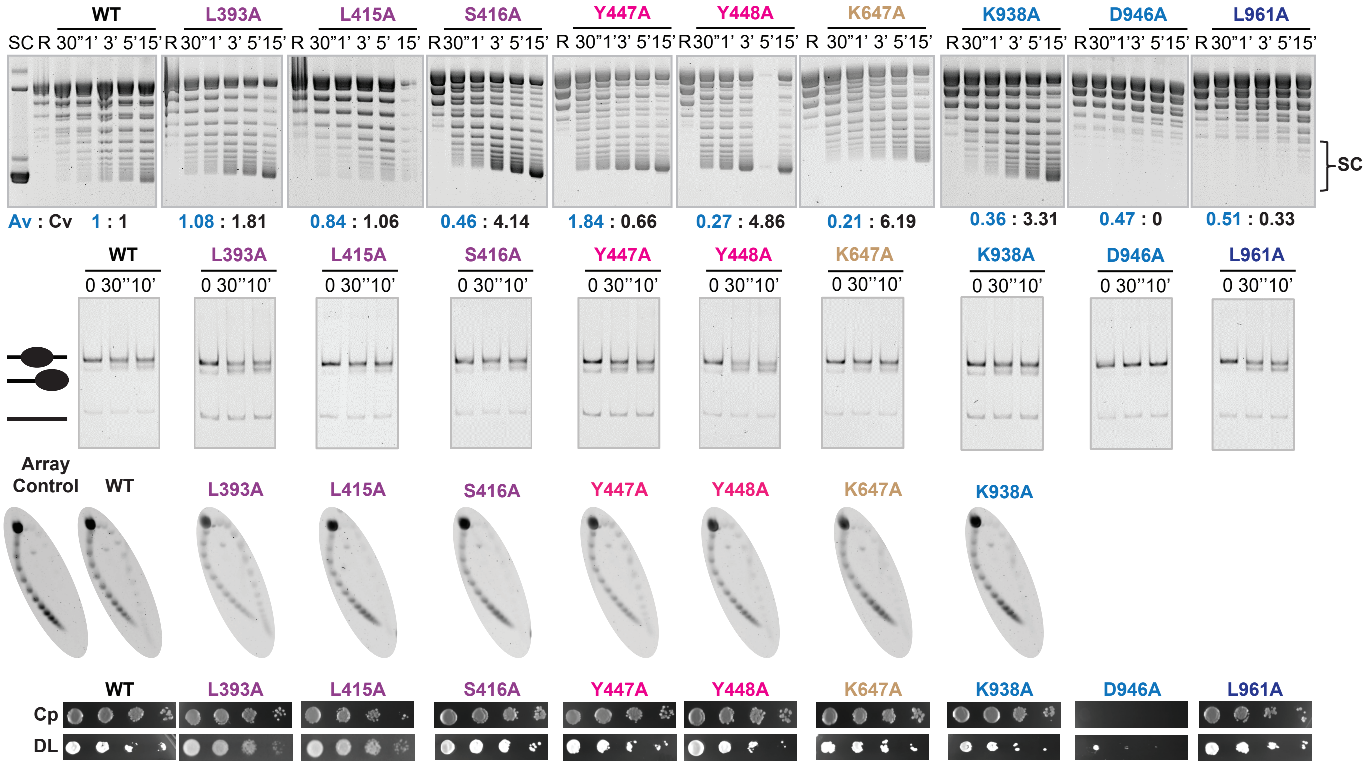

B

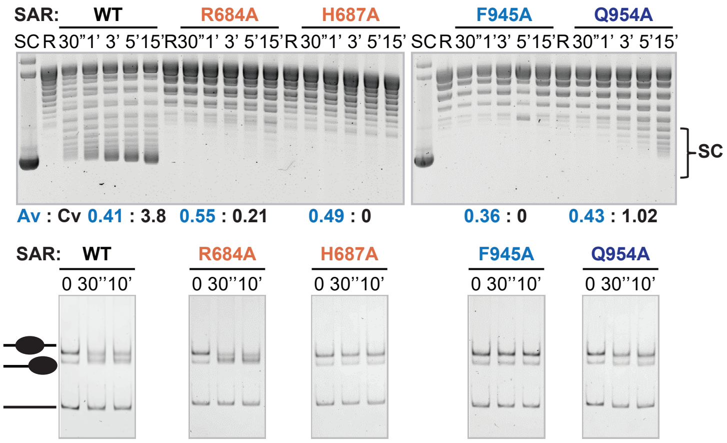

(A) Comparative impact of additional alanine substitutions either enhancing or abolishing DNA translocation, coupling, and nucleosome sliding, confirming two distinct functional regions within the hub. All assays performed and results depicted as in Figure 2A.

(B) First row: Comparative DNA translocation and ATPase activity by Sth1 derivatives in the SAR format. Addition of the ARP module to Sth1 (SAR) enhances DNA translocation for mutations R684A and Q954A, but not H687A and F945A. The plasmid stimulated ATPase activity/value (Av, blue text) and coupling value (Cv, black text; expressed as supercoiling/ATPase hydrolyzed) and normalized to WT Sth1 alone (Av:Cv=1:1). SC: highly-supercoiled topoisomers, R: relaxed plasmid.
Second row: Comparative sliding of mononucleosomes performed using SAR derivatives. Addition of the ARP module to Sth1 (SAR) enhances nucleosome sliding for mutations R684A and Q954A, but not H687A and F945A.

Figure S3, related to Figures 3 and 4.
A scatter plot comparing the biochemical properties (ATPase values and Coupling values) of Sth1 mutations groups/clusters the mutants into three categories: loss-of-function, gain-of-function and dominant lethal.

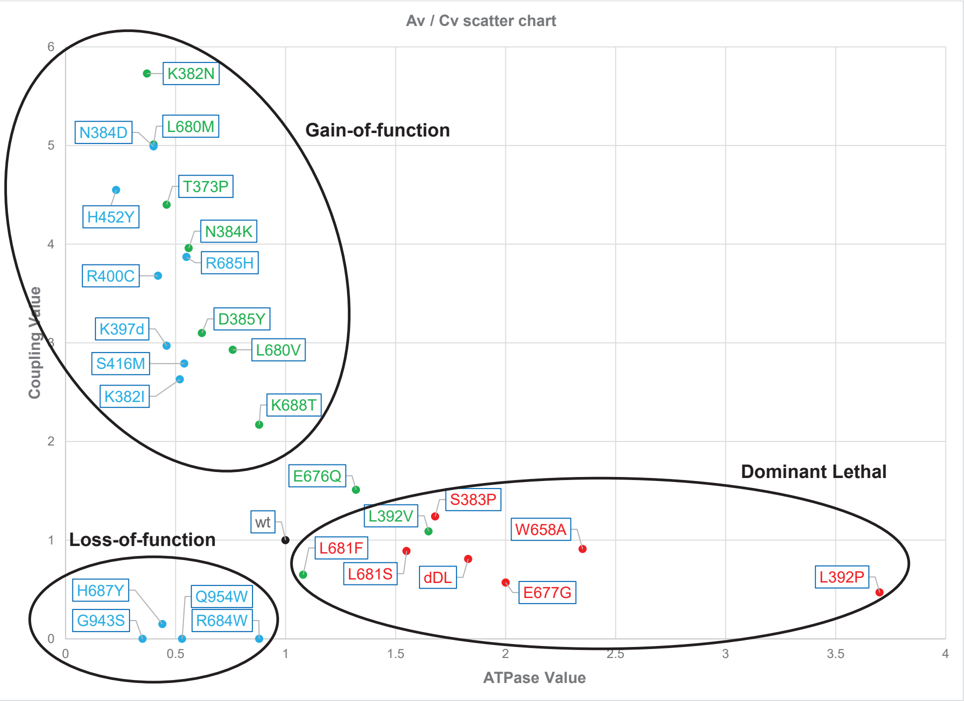

Scatter plot of the ATPase / Coupling values from various mutations. Cancer-associated mutations are depicted with blue markers and labels. Mutations compensating for the lack of the ARPs (*mra*), and not dominant lethal, are depicted with green markers and labels. Mutation L681F, also compensating for the lack of the ARPs but dominant lethal, is depicted with a green marker, but red label. Mutations that are dominant-lethal and are unable to complement *sth1*Δ, including W658A, are depicted with a red marker and label. Notably, cancer-associated mutations partitioned into two distinct clusters corresponding to the loss-of-function and the gain-of-function mutations: a first cluster characterized by a lowering of the ATPase activity and a loss of coupling when compared to wild-type (wt; ATPase and coupling values both normalized to 1.0 for wild-type) grouped the loss-of-function cancer-associated mutations. In contrast, a second cluster, while still displaying reduced ATPase activity, presented an increase in coupling and grouped the gain-of-function cancer-associated mutations. Of note, the vast majority of the *mra* mutations belonged to this cluster. Finally, a third cluster characterized by an increase of the ATPase activity without change in coupling, contained the mutations that are dominant-lethal. Remarkably, the three clusters defined here by the scatter plot of the ATPase and coupling values matched the three clusters generated by the hierarchical clustering of the ATAC-seq samples (Figure 5).

Figure S4, related to Figure 5.
ATAC-seq Accessibility, Mutant Sth1 Incorporation, and Heat Map.

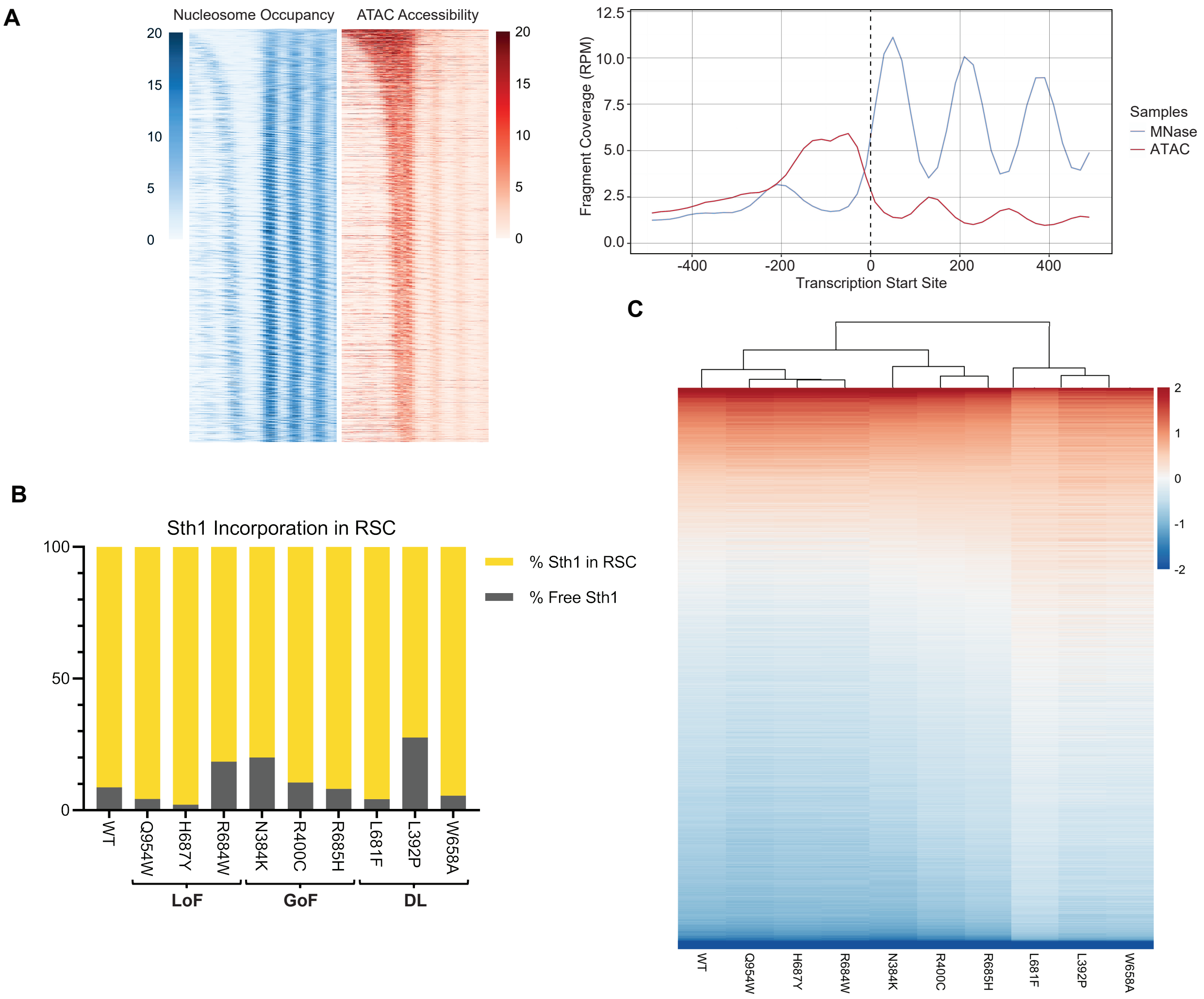

(A) **Nucleosome occupancy comparisons to ATAC accessibility.** Heat maps of signal from wild type strains around the TSS for protein-coding genes (rows), ordered by the decreasing length of the promoter NDR. The nucleosome occupancy represents the signal from MNase fragment coverage (recording the central 75 bp of each fragment). The signal indicates strong (dark blue) or weak (light blue) nucleosome occupancy. The ATAC accessibility represents the fragment coverage (20 bp) encompassing each observed ATAC cut site. The signal indicates the strong (dark red) or weak (light red) accessibility of DNA not wrapped in nucleosome and/or proteins. On the right is a graph of the mean profile signal density for both nucleosome occupancy and ATAC-seq.

(B) **Mutant Sth1 incorporation in RSC complex.** *S. cerevisiae* cells were transformed and grown as described in methods for ATAC-seq, cells were lysed, and the whole cell extract immunoprecipitaed with α-Flag resin. The bound fraction was eluted, run on a non-denaturing protein gel (Invitrogen NativePAGE Bis-Tris Gel System), and probed with α-FLAG to detect the slow migrating RSC (with mutant Sth1 incorporated) and the faster migrating free mutant Sth1 subunit present in the whole cell lysate. The bands intensities corresponding to slow migrating RSC and faster-migrating free Sth1 were quantified using ImageJ. The percentage of free Sth1 (in gray) and Sth1 incorporated into RSC (in yellow) for WT and each mutant were plotted as a bar graph.

(C) **Mean accessibility heat map over 1 kb of all TSS, and derived hierarchical clustering tree.** Log2 fold ratio of signal over background was collected in a 1 kb window (-500 to +500 bp) of protein-coding gene TSS and plotted as a heat map.

Figure S5, related to Figure 5.
Gallery of illustrative snapshots from a genome browser.

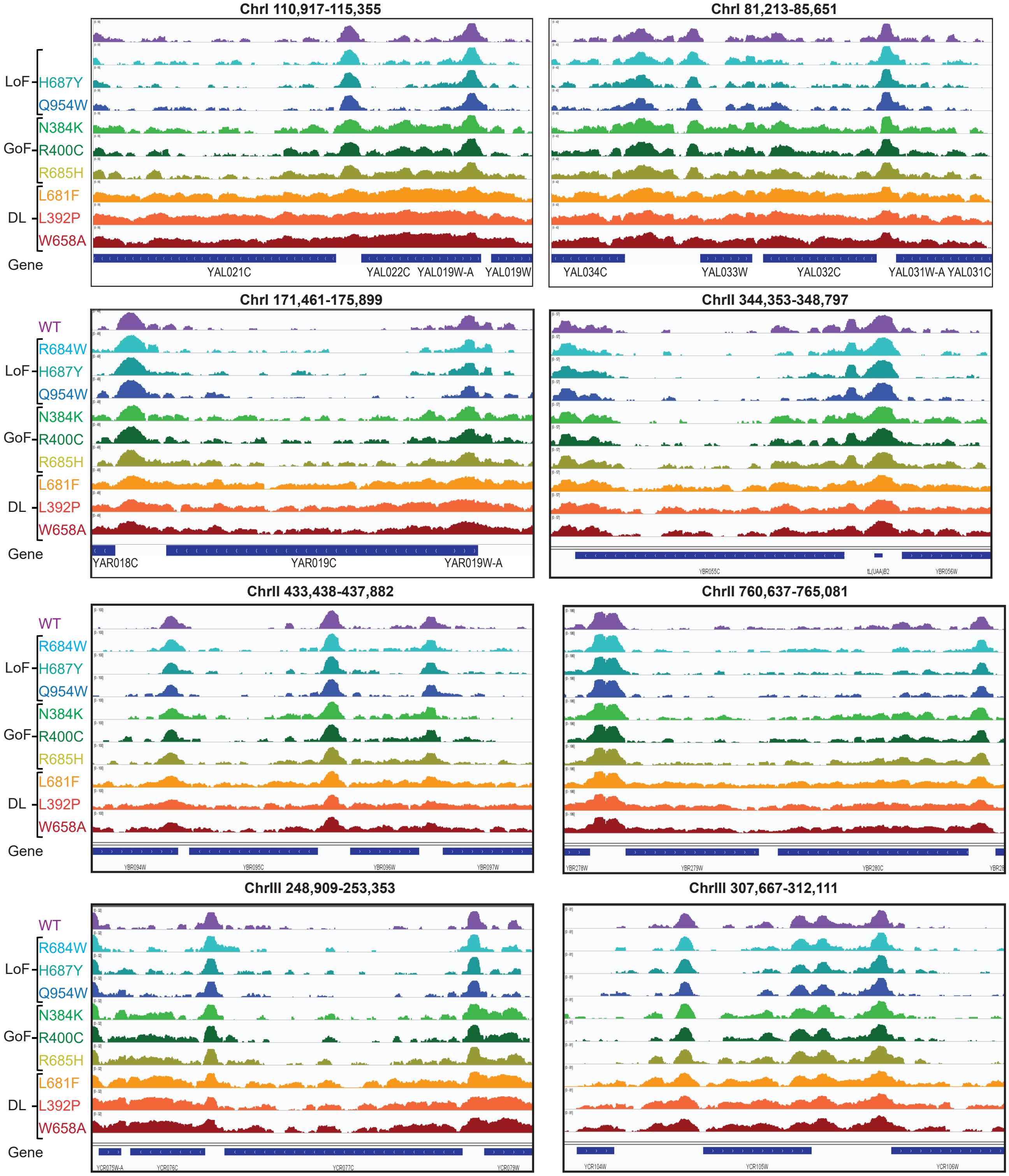

Snapshots from a genome browser showing the ATAC-seq accessibility coverage (Log RPM) across the chromosome. All tracks within each snapshot are scaled to the same Y axis. Progressive/increasing impact is generally observed as one examines the loss-of-function, gain-of-function cancer-associated missense, and dominant lethal mutations.

Figure S6, related to Figure 6.
In the context of the Snf2 ATPase alone (lacking the ARP module and other subunits), the position of most of the hub domains are shifted, along with a 3' to 5' one base-pair shift in the positioning of the ATPase lobe 2 with respect to lobe 1, opening the ATP binding/hydrolysis cleft, revealing a possible intermediate conformation in the DNA translocation mechanism.

A

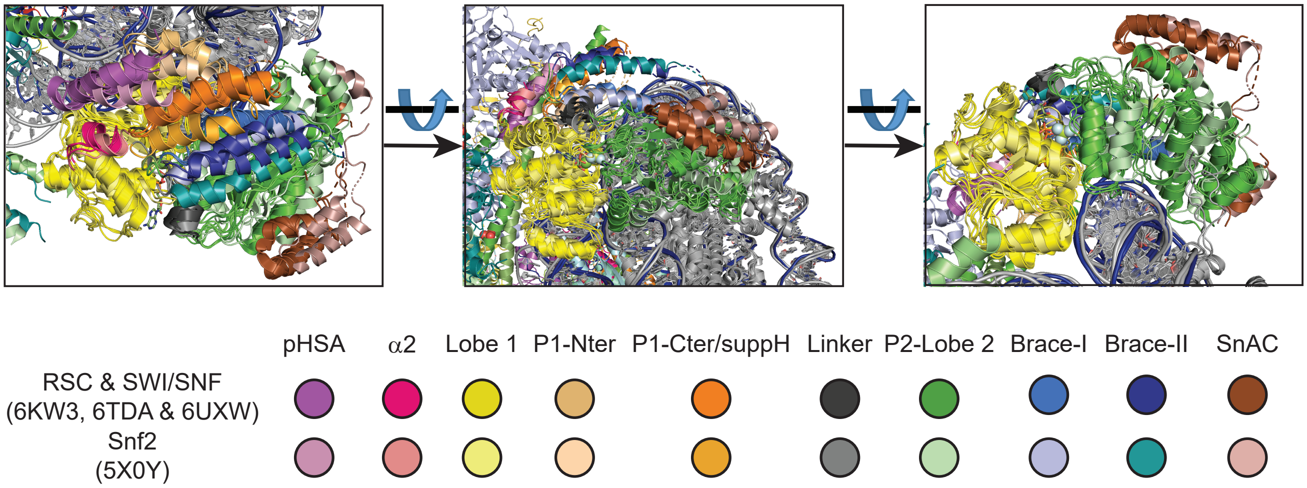

B

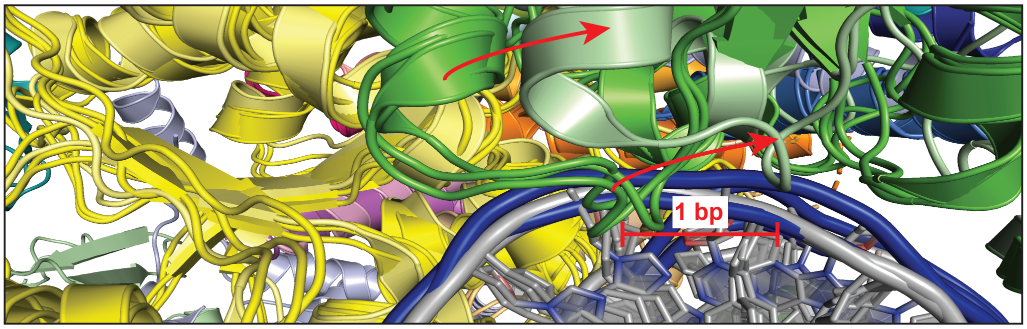

(A) Structural alignment of various Remodeler-nucleosome complexes (PDB ID 5X0Y, 6KW3, 6TDA and 6UXW) based on the alignment of their respective histone octamers reveals that, in the absence of the ARP module and other subunits, there is a structural shift of many hub domains, along with a structural shift of the ATPase lobe 2 away from lobe 1. The different views represent snapshots on a sequential rotation of the initial alignment on the left. Domains of the RSC & SWI/SNF Remodeler-nucleosome complexes (PDB ID 6KW3, 6TDA, 6UXW) and the Snf2-nucleosome complex (PDB ID5X0Y) are color-coded according to the depicted entry key below. Structural alignment has been obtained using The PyMOL Molecular Graphics System, Version 2.3.2 Schrödinger, LLC.
(B) Magnification of the right view of panel (A) showing that the shift in positioning of lobe 2 with respect to lobe 1 (red arrows) matches the distance of one base-pair along the bound DNA (blind-ended red line), revealing a possible intermediate conformation in the DNA translocation mechanism. Domains are color-coded as in (A).

Table S1, related to Figures 2, 3, 4, 5, and 6.

All mutations investigated with biochemistry, genetic and genomics data presented together along with functional classification.

Hub Domain	Sth1 Residue & Mutation	DNA Translocation	ATPase value	Coupling value	Mono-nuc. Sliding	Array-nuc. Ejection	Genet. Cp	Genet. DL	ATAC-seq	GoF, LoF. & DL	Hub Region
HSA-Cter	T373P	✓↑	↓	↑↑	?	?	✓	×		GoF	#1
	K382N	✓↑	↓	↑↑	?	?	✓	×		GoF	#1
	K382I	✓↑	↓	↑↑	✓↑	✓	✓	×		GoF	#1
	S383P	✓↑	↑	↑	?	?	×	✓		GoF+DL	#1
	N384K	✓↑	↓	↑↑	?	?	✓	×	✓↑	GoF	#1
	N384D	✓↑	↓	↑↑	✓↑	✓	✓	×		GoF	#1
	D385Y	✓↑	↓	↑↑	?	?	✓	✓		GoF	#1
	L392V	✓↑	↑	=	✓ (*)	✓↑ (*)	✓	× (*)		GoF	#1
	L392P	✓↑	↑↑	↓↓	✓ (*)	✓↑↑ (*)	×	✓ (*)	✓↑↑	GoF+DL	#1
	ΔDL	✓↑	↑	↓	✓ (*)	✓↑ (*)	×	✓ (*)		GoF+DL	#1
pHSA	L393A	✓↑	=	↑	✓↑	✓↑	✓	×		GoF	#1
	D394A	✓↑	↓	↑↑	✓↑	✓	✓	×		GoF	#1
	K397A	✓↑	↓	↑↑	✓↑	×	✓	×		GoF	#1
	K397Δ	✓↑	↓	↑↑	✓↑	✓	×	×		GoF	#1
	R400A	✓↑	↓	↑↑	✓↑	✓	✓	×		GoF	#1
	R400C	✓↑	↓	↑↑	✓↑	✓	✓	×	✓↑	GoF	#1
	L415A	✓=	↓	=	✓=	✓	✓	×		GoF	#1
	S416A	✓↑	↓	↑↑	✓=	×	✓	×		GoF	#1
	S416M	✓↑	↓	↑↑	✓↑	✓	✓	×		GoF	#1
α2	Y447A	✓↑	↑	↓	✓↑	✓↑	✓	×		GoF	#1
	Y448A	✓↑	↓↓	↑↑	✓↑	✓	✓	×		GoF	#1
	H452A	✓↑	↓↓	↑↑	✓=	×	✓	×		GoF	#1
	H452Y	✓=	↓↓	↑↑	✓↑	✓	✓	×		GoF	#1
P1-Nter	K647A	✓=	↓↓	↑↑	✓=	✓	✓	×		GoF	#1
	F655A	✓=	↓	↑	✓↑↑	✓	✓	×		GoF	#1
	W658A	✓↑	↑	=	✓↑↑	✓↑↑	×	✓	✓↑↑	GoF+DL	#1
	F659A	✓↑	↓	↑	✓↑↑	✓↑	✓	✓		GoF	#1
P1-Cter / suppH	E676Q	✓↑	↑	↑	✓ (*)	✓↑ (*)	✓	× (3)		GoF	#1
	E677G	✓=	↑↑	↓	?	?	×	✓		GoF+DL	#1
	L680M	✓↑	↓	↑↑	?	?	✓	×		GoF	#1
	L680V	✓↑	↓	↑↑	?	?	✓	×		GoF	#1
	L681F	✓=	=	↓	✓ (*)	✓ (*)	✓	✓ (3)	✓↑↑	GoF+DL	#1
	L681S	✓↑	↑	↓	?	?	✓	✓		GoF+DL	#1
	R684A	× (1)	↓	~0	× (1)	×	✓	×		LoF	#2
	R684W	×	↓	0	×	×	×	×	✓=	LoF	#2
	R685A	✓↑	↓	↑	✓↑	✓↑	✓	×		GoF	#1
	R685H	✓↑	↓	↑↑	✓↑	✓↑	✓	×	✓↑	GoF	#1
	H687A	× (2)	↓	0	× (2)	×	×	✓		LoF	#2
	H687Y	×	↓↓	~0	×	×	×	✓	✓=	LoF	#2
	K688A	✓↑	↓	↑↑	✓=	×	✓	×		GoF	#1
	K688T	✓↑	↓	↑↑	?	?	✓	×		GoF	#1
	R691A	×	↓	0	✓↓	×	✓	×		LoF	#2
Brace-I	K938A	✓↑	↓	↑↑	✓↑	✓	✓	×		GoF	#1
	V939A	×	↓	0	×	×	✓	×		LoF	#2
	G943S	×	↓↓	0	×	×	×	✓		LoF	#2
	F945A	× (2)	↓	0	× (2)	×	×	✓		LoF	#2
	D946A	×	↓	0	×	?	×	✓		LoF	#2
Brace-II	Q954A	× (1)	↓	↓	× (1)	✓	✓	×		LoF	#2
	Q954W	×	↓	0	×	×	×	×	✓=	LoF	#2
	L958A	×	↓	0	×	×	✓	×		LoF	#2
	L961A	×	↓	↓↓	✓↑	?	✓	×		LoF	#2

The legend for this table is located on the next page.

In biochemistry assays: mutant does (✓), or does not (×), translocate DNA, slide mono-nucleosomes, or eject nucleosomes from arrays. Activities: =, ↑, ↑↑, ↓, ↓↓: Equal, Moderate or strong, increase or decrease, when compared to WT; 0: No activity; ?: untested.

In genetic experiments: In complementation assays (Genet. Cp), mutant fully (✓), or weakly (✓) complements, or does not complement (×). In dominant-lethality assay (Genet. DL), mutant is dominant lethal (✓), or dominant slow growing (✓), or not impacted (×).

In genomics, ATAC-seq performed (✓), with chromatin accessibility equal (=), moderately (↑) or strongly (↑↑) increased when compared to WT.

Functional classification: GoF = gain-of-function, LoF = loss-of-function, DL = dominant-lethality.

(1): ✓ if in the SAR format, contrasted to: (2): × if in the SAR format.
(*): Experiments documented in Clapier et al., 2016.

Meaning of the shading color, and location in the Results:

	Region #1 alanine substitutions, described in “Alanine Scanning Mutations”
	Region #1 alanine substitutions displaying increased nucleosome ejection, described in “Alanine Scanning Mutations”
	Region #2 alanine substitutions, described in “Alanine Scanning Mutations”
	Region #2 alanine substitutions requiring ARP module restoration, described in “Alanine Scanning Mutations”
	mra mutations, described in “Mutations that Suppress *arp*Δ Mutations”
	Dominant lethal mutations, described in “Mutations that Suppress *arp*Δ Mutations”
	Gain-of-function Cancer-Associated Missense Mutations, described in “Gain-of-function Cancer-Associated Mutations”’
	Loss-of-function Cancer-Associated Missense Mutations, described in “Loss-of-function Cancer-Associated Mutations”’
	Region #1, regulatory region of the SWI/SNF structural hub
	Region #2, implementation region of the SWI/SNF structural hub

Table S2, related to Key Resources Table.

Oligonucleotides used for site-directed mutagenesis and sequencing.

Oligo Name	Oligo Sequence	Source
CC_SDM373PF	GATGAACAGAAAAGAATAGAAAGACCGGCTAAGCAACGTTTAGCTGCTTTG	This paper
CC_SDM373PR	CAAAGCAGCTAAACGTTGCTTAGCCGGTCTTTCTATTCTTTTCTGTTCATC	This paper
CC_SDM382NF	GCAACGTTTAGCTGCTTTGAACTCTAACGATGAGGAGGCTTATTTAAAG	This paper
CC_SDM382NR	CTTTAAATAAGCCTCCTCATCGTTAGAGTTCAAAGCAGCTAAACGTTGC	This paper
CC_SDM382IF2	GCTAAGCAACGTTTAGCTGCTTTGATTTCTAACGATGAGGAGGCTTATTTAAAGC	This paper
CC_SDM382IR2	GCTTTAAATAAGCCTCCTCATCGTTAGAAATCAAAGCAGCTAAACGTTGCTTAGC	This paper
CC_SDM383PF	GCAACGTTTAGCTGCTTTGAAGCCGAACGATGAGGAGGCTTATTTAAAG	This paper
CC_SDM383PR	CTTTAAATAAGCCTCCTCATCGTTCGGCTTCAAAGCAGCTAAACGTTGC	This paper
CC_SDM384KF	GCAACGTTTAGCTGCTTTGAAGTCTAAAGATGAGGAGGCTTATTTAAAG	This paper
CC_SDM384KR	CTTTAAATAAGCCTCCTCATCTTTAGACTTCAAAGCAGCTAAACGTTGC	This paper
CC_SDM384DF	GCAACGTTTAGCTGCTTTGAAGTCTGACGATGAGGAGGCTTATTTAAAG	This paper
CC_SDM384DR	CTTTAAATAAGCCTCCTCATCGTCAGACTTCAAAGCAGCTAAACGTTGC	This paper
CC_SDM385YF	CGTTTAGCTGCTTTGAAGTCTAACTATGAGGAGGCTTATTTAAAGCTTTTAG	This paper
CC_SDM385YR	CTAAAAGCTTTAAATAAGCCTCCTCATAGTTAGACTTCAAAGCAGCTAAACG	This paper
CC_SDM393AF	GATGAGGAGGCTTATTTAAAGCTTGCGGATCAAACTAAGGATACTAG	This paper
CC_SDM393AR	CTAGTATCCTTAGTTTGATCCGCAAGCTTTAAATAAGCCTCCTCATC	This paper
CC_SDM394AF	GAGGAGGCTTATTTAAAGCTTTTAGCGCAAACTAAGGATACTAGAATTAC	This paper
CC_SDM394AR	GTAATTCTAGTATCCTTAGTTTGCGCTAAAAGCTTTAAATAAGCCTCCTC	This paper
CC_SDM397AF	CTTATTTAAAGCTTTTAGATCAAACTGCGGATACTAGAATTACACAGTTATTG	This paper
CC_SDM397AR	CAATAACTGTGTAATTCTAGTATCCGCAGTTTGATCTAAAAGCTTTAAATAAG	This paper
CC_SDMK397delF	GGCTTATTTAAAGCTTTTAGATCAAACTGATACTAGAATTACACAGTTATTGAGGC	This paper
CC_SDMK397delR	GCCTCAATAACTGTGTAATTCTAGTATCAGTTTGATCTAAAAGCTTTAAATAAGCC	This paper
CC_SDM400AF	GCTTTTAGATCAAACTAAGGATACTGCGATTACACAGTTATTGAGGCAGAC	This paper
CC_SDM400AR	GTCTGCCTCAATAACTGTGTAATCGCAGTATCCTTAGTTTGATCTAAAAGC	This paper
CC_SDM400CF	GCTTTTAGATCAAACTAAGGATACTTGCATTACACAGTTATTGAGGCAGAC	This paper
CC_SDM400CR	GTCTGCCTCAATAACTGTGTAATGCAAGTATCCTTAGTTTGATCTAAAAGC	This paper
CC_SDM415AF	GACTAATTCCTTTTTGGATTCTGCGTCGGAGGCTGTTAGAGCACAAC	This paper
CC_SDM415AR	GTTGTGCTCTAACAGCCTCCGACGCAGAATCCAAAAAGGAATTAGTC	This paper
CC_SDM416AF	GACTAATTCCTTTTTGGATTCTTTGGCGGAGGCTGTTAGAGCACAACAAAAC	This paper
CC_SDM416AR	GTTTTGTTGTGCTCTAACAGCCTCCGCCAAAGAATCCAAAAAGGAATTAGTC	This paper
CC_SDMS416MF	GACTAATTCCTTTTTGGATTCTTTGATGGAGGCTGTTAGAGCACAACAAAACG	This paper
CC_SDMS416MR	CGTTTTGTTGTGCTCTAACAGCCTCCATCAAAGAATCCAAAAAGGAATTAGTC	This paper
CC_SDM447AF	GATGAAGAAAGAGAAAAGACTGACGCGTATGAAGTCGCTCATAGGATCAAAG	This paper
CC_SDM447AR	CTTTGATCCTATGAGCGACTTCATACGCGTCAGTCTTTTCTCTTTCTTCATC	This paper
CC_SDM448AF	GATGAAGAAAGAGAAAAGACTGACTATGCGGAAGTCGCTCATAGGATCAAAG	This paper
CC_SDM448AR	CTTTGATCCTATGAGCGACTTCCGCATAGTCAGTCTTTTCTCTTTCTTCATC	This paper
CC_SDM452AF	GACTGACTATTATGAAGTCGCTGCGAGGATCAAAGAAAAAATTGATAAAC	This paper
CC_SDM452AR	GTTTATCAATTTTTTCTTTGATCCTCGCAGCGACTTCATAATAGTCAGTC	This paper
CC_SDMH452YF	GACTGACTATTATGAAGTCGCTTATAGGATCAAAGAAAAAATTGATAAACAACC	This paper
CC_SDMH452YR	GGTTGTTTATCAATTTTTTCTTTGATCCTATAAGCGACTTCATAATAGTCAGTC	This paper
CC_SDM647AF	GCACTGTTGAACTTTGTTTTGCCTGCGATTTTCAATTCTGCGAAGACTTTTG	This paper
CC_SDM647AR	CAAAAGTCTTCGCAGAATTGAAAATCGCAGGCAAAACAAAGTTCAACAGTGC	This paper
CC_SDM655AF	GCCTAAAATTTTCAATTCTGCGAAGACTGCGGAAGATTGGTTTAATACTCC	This paper
CC_SDM655AR	GGAGTATTAAACCAATCTTCCGCAGTCTTCGCAGAATTGAAAATTTTAGGC	This paper
CC_SDM658AF	CAATTCTGCGAAGACTTTTGAAGATGCGTTTAATACTCCATTTGCCAACACCG	This paper
CC_SDM658AR	CGGTGTTGGCAAATGGAGTATTAAACGCATCTTCAAAAGTCTTCGCAGAATTG	This paper
CC_SDM659AF	CTGCGAAGACTTTTGAAGATTGGGCTAATACTCCATTTGCCAACACCGG	This paper
CC_SDM659AR	CCGGTGTTGGCAAATGGAGTATTAGCCCAATCTTCAAAAGTCTTCGCAG	This paper
CC_SDM680MF	CTAGAATTAACGGAAGAAGAAACTATGTTAATTATTAGAAGATTACATAAG	This paper
CC_SDM680MR	CTTATGTAATCTTCTAATAATTAACATAGTTTCTTCTTCCGTTAATTCTAG	This paper
CC_SDM680VF	CTAGAATTAACGGAAGAAGAAACTGTGTTAATTATTAGAAGATTACATAAG	This paper
CC_SDM680VR	CTTATGTAATCTTCTAATAATTAACACAGTTTCTTCTTCCGTTAATTCTAG	This paper
CC_SDM684AF	CGGAAGAAGAAACTCTGTTAATTATTGCGAGATTACATAAGGTGTTAAGGCC	This paper
CC_SDM684AR	GGCCTTAACACCTTATGTAATCTCGCAATAATTAACAGAGTTTCTTCTTCCG	This paper
CC_SDM684WF	CGGAAGAAGAAACTCTGTTAATTATTTGGAGATTACATAAGGTGTTAAGGCC	This paper

CC_SDM684WR	GGCCTTAACACCTTATGTAATCTCCAAATAATTAACAGAGTTTCTTCTTCCG	This paper
CC_SDM685AF	GAAACTCTGTTAATTATTAGAGCGTTACATAAGGTGTTAAGGCCA	This paper
CC_SDM685AR	TGGCCTTAACACCTTATGTAACGCTCTAATAATTAACAGAGTTTC	This paper
CC_SDM685HF	GAAACTCTGTTAATTATTAGACATTTACATAAGGTGTTAAGGCCA	This paper
CC_SDM685HR	TGGCCTTAACACCTTATGTAAATGTCTAATAATTAACAGAGTTTC	This paper
CC_SDM687AF	GAAACTCTGTTAATTATTAGAAGATTAGCGAAGGTGTTAAGGCCATTTTTA	This paper
CC_SDM687AR	TAAAAATGGCCTTAACACCTTCGCTAATCTTCTAATAATTAACAGAGTTTC	This paper
CC_SDM687YF2	CTCTGTTAATTATTAGAAGATTATATAAGGTGTTAAGGCCATTTTTATTGCGTCG	This paper
CC_SDM687YR2	CGACGCAATAAAAATGGCCTTAACACCTTATATAATCTTCTAATAATTAACAGAG	This paper
CC_SDM688AF	CTCTGTTAATTATTAGAAGATTACATGCGGTGTTAAGGCCATTTTTATTGCG	This paper
CC_SDM688AR	CGCAATAAAAATGGCCTTAACACCGCATGTAATCTTCTAATAATTAACAGAG	This paper
CC_SDM688TF	CTGTTAATTATTAGAAGATTACATACCGTGTTAAGGCCATTTTTATTGCG	This paper
CC_SDM688TR	CGCAATAAAAATGGCCTTAACACGGTATGTAATCTTCTAATAATTAACAG	This paper
CC_SDM691AF	TTATTAGAAGATTACATAAGGTGTTAGCGCCATTTTTATTGCGTCGTTTG	This paper
CC_SDM691AR	CAAACGACGCAATAAAAATGGCGCTAACACCTTATGTAATCTTCTAATAA	This paper
CC_SDM938AF	GCAGAAATTGGACATTGACGGCGCGGTTATCCAGGCAGGTAAGTTTGATAAC	This paper
CC_SDM938AR	GTTATCAAACTTACCTGCCTGGATAACCGCGCCGTCAATGTCCAATTTCTGC	This paper
CC_SDM939AF	GAAATTGGACATTGACGGCAAGGCGATCCAGGCAGGTAAGTTTGATAACAAATC	This paper
CC_SDM939AR	GATTTGTTATCAAACTTACCTGCCTGGATCGCCTTGCCGTCAATGTCCAATTTC	This paper
CC_SDM943SF	CATTGACGGCAAGGTTATCCAGGCAAGCAAGTTTGATAACAAATCAACTGCAG	This paper
CC_SDM943SR	CTGCAGTTGATTTGTTATCAAACTTGCTTGCCTGGATAACCTTGCCGTCAATG	This paper
CC_SDM945AF	GGCAAGGTTATCCAGGCAGGTAAGGCGGATAACAAATCAACTGCAGAAGAG	This paper
CC_SDM945AR	CTCTTCTGCAGTTGATTTGTTATCCGCCTTACCTGCCTGGATAACCTTGCC	This paper
CC_SDM946AF	CAAGGTTATCCAGGCAGGTAAGTTTGCGAACAAATCAACTGCAGAAGAGCAAG	This paper
CC_SDM946AR	CTTGCTCTTCTGCAGTTGATTTGTTCGCAAACTTACCTGCCTGGATAACCTTG	This paper
CC_SDM954AF	GATAACAAATCAACTGCAGAAGAGGCGGAAGCTTTTTTGAGAAGATTG	This paper
CC_SDM954AR	CAATCTTCTCAAAAAAGCTTCCGCCTCTTCTGCAGTTGATTTGTTATC	This paper
CC_SDM954WF	GATAACAAATCAACTGCAGAAGAGTGGGAAGCTTTTTTGAGAAGATTG	This paper
CC_SDM954WR	CAATCTTCTCAAAAAAGCTTCCCACTCTTCTGCAGTTGATTTGTTATC	This paper
CC_SDM958AF	GCAGAAGAGCAAGAAGCTTTTGCGAGAAGATTGATCGAAAGTGAAACG	This paper
CC_SDM958AR	CGTTTCACTTTCGATCAATCTTCTCGCAAAAGCTTCTTGCTCTTCTGC	This paper
CC_SDM961AF	GAGCAAGAAGCTTTTTTGAGAAGAGCGATCGAAAGTGAAACGAACAGG	This paper
CC_SDM961AR	CCTGTTCGTTTCACTTTCGATCGCTCTTCTCAAAAAAGCTTCTTGCTC	This paper
CC_TR3Fseq	CATACTGCCGCCCTGACCGAC	This paper
CC_STH1R03	GTAATTCAAACTTGCCCGCTACTCTG	This paper
CC_STH1BsFseq	CTAGAGCCGGTGGTTTGGGTTTG	This paper

Note: L392V, L392P, ΔDL, E676Q, E677G, L681F, and L681S constructs were derived from plasmids used in previous published (Clapier et al., 2016) and unpublished studies, and will be provided upon request.
