# Supplementary material for: Cancer-Associated Gain-of-Function Mutations Activate a SWI/SNF-Family Regulatory Hub
Source: Mol Cell. 2020 Nov 19;80(4):712–725.e5. doi: 10.1016/j.molcel.2020.09.024 (PMC7853424; doi:10.1016/j.molcel.2020.09.024)
Supplement: Document S2. Article plus Supplemental Information [file mmc2.pdf]

Molecular Cell

Article

Cancer-Associated Gain-of-Function Mutations Activate a SWI/SNF-Family Regulatory Hub

Graphical Abstract

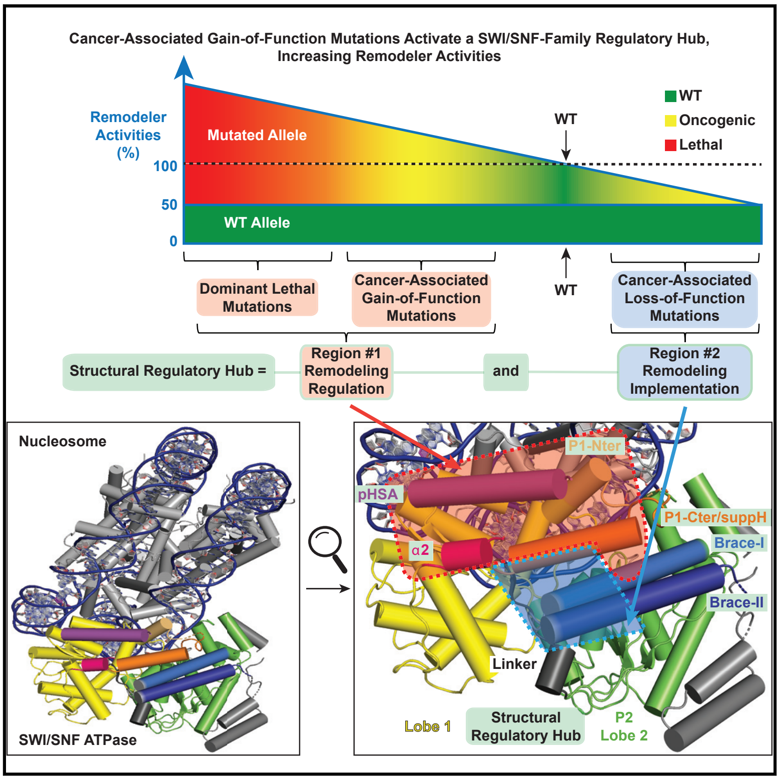

Authors

Cedric R. Clapier, Naveen Verma, Timothy J. Parnell, Bradley R. Cairns

Correspondence

cedric.clapier@hci.utah.edu (C.R.C.), brad.cairns@hci.utah.edu (B.R.C.)

In Brief

Nucleosome sliding by SWI/SNF-family remodelers involves regulated ATP-dependent DNA translocation. Here, Clapier, Verma et al. reveal a conserved bi-partite integrative regulatory hub within SWI/SNF-family ATPases. Region 1 regulates DNA translocation efficiency and harbors gain-of-function cancer-associated mutations, which increase sliding and chromatin accessibility, whereas region 2 implements DNA translocation, providing structure-function mechanistic insight.

Highlights

- Domains flanking and linking the ATPase lobes form a conserved structural hub
- The hub has two distinct regions, to either regulate or implement DNA translocation
- Cancer-associated GoF and LoF missense mutations map separately to each region
- Only GoF mutations increase nucleosome sliding and chromatin accessibility

Clapier et al., 2020, Molecular Cell *80*, 712–725
November 19, 2020 © 2020 Elsevier Inc.
https://doi.org/10.1016/j.molcel.2020.09.024

CellPress

CellPress

Molecular Cell

Article

Cancer-Associated Gain-of-Function Mutations Activate a SWI/SNF-Family Regulatory Hub

Cedric R. Clapier,[1,2,3,*] Naveen Verma,[1,2,3] Timothy J. Parnell,[2] and Bradley R. Cairns[1,2,4,*]
[1]Department of Oncological Sciences and Howard Hughes Medical Institute, University of Utah School of Medicine, Salt Lake City, UT 84112, USA
[2]Huntsman Cancer Institute, University of Utah School of Medicine, Salt Lake City, UT 84112, USA
[3]These authors contributed equally
[4]Lead Contact
*Correspondence: cedric.clapier@hci.utah.edu (C.R.C.), brad.cairns@hci.utah.edu (B.R.C.)
https://doi.org/10.1016/j.molcel.2020.09.024

SUMMARY

SWI/SNF-family remodelers (BAF/PBAF in mammals) are essential chromatin regulators, and mutations in human BAF/PBAF components are associated with ∼20% of cancers. Cancer-associated missense mutations in human *BRG1* (encoding the catalytic ATPase) have been characterized previously as conferring loss-of-function. Here, we show that cancer-associated missense mutations in *BRG1*, when placed into the orthologous Sth1 ATPase of the yeast RSC remodeler, separate into two categories: loss-of-function enzymes, or instead, gain-of-function enzymes that greatly improve DNA translocation efficiency and nucleosome remodeling *in vitro*. Our work identifies a structural "hub," formed by the association of several Sth1 domains, that regulates ATPase activity and DNA translocation efficiency. Remarkably, all gain-of-function cancer-associated mutations and all loss-of-function mutations physically localize to distinct adjacent regions in the hub, which specifically regulate and implement DNA translocation, respectively. *In vivo*, only gain-of-function cancer-associated mutations conferred precocious chromatin accessibility. Taken together, we provide a structure-function mechanistic basis for cancer-associated hyperactivity.

INTRODUCTION

Chromatin remodeling involves the use of ATP-dependent complexes, termed remodelers, to move, restructure, and/or eject nucleosomes—the basic unit of chromatin structure (Becker and Workman, 2013; Clapier and Cairns, 2014; Clapier et al., 2017). Chromatin remodelers are a diverse set of complexes that are utilized in a variety of nuclear processes (Clapier and Cairns, 2014; Narlikar et al., 2013). Remodelers occupy gene enhancers, promoters, and coding regions to regulate nucleosome occupancy and positioning, and therefore impact the ability of transcription factors and regulators to bind DNA within chromatin (Clapier and Cairns, 2014; Lorch and Kornberg, 2015). Proper transcriptional regulation is important for establishing and maintaining cellular identity, and by affecting dynamic chromatin accessibility, remodelers impact developmental potential and proliferation (Ho and Crabtree, 2010).

Notably, mutations in subunits of remodelers are present in ∼20% of human cancers and in a wide array of specific developmental disorders/syndromes (Helming et al., 2014; Kadoch et al., 2013; Shain and Pollack, 2013; Sokpor et al., 2017). More specifically, SWI/SNF-remodeler mutations are found at high frequency in malignant rhabdoid tumors (>95%), ovarian clear cell carcinoma (75%), clear cell renal carcinoma (57%), hepatocellular carcinoma (40%), gastric cancer (36%), melanoma (34%), and pancreatic cancer (26%). Of note, tumors with SWI/SNF mutations typically contain very few additional genetic mutations, suggesting that mutations in the remodelers have the potential to be driver mutations by providing a decisive advantage for tumor initiation or growth (Dutta et al., 2017; Helming et al., 2014; Shain and Pollack, 2013; Wilson and Roberts, 2011). Importantly, mutations in the catalytic ATPase-DNA translocase subunit of BAF/PBAF, named BRG1 in humans, tend to be heterozygous in cancers, suggesting that they are either haploinsufficient or act through dominant mechanisms such as gain-of-function or dominant-negative interference. Loss-of-function mutations in SWI/SNF-family remodelers (lacking remodeling activity) have been described previously (Bultman et al., 2005; Dykhuizen et al., 2013; Hodges et al., 2018); however, a key unanswered question is whether gain-of-function mutations exist—and if so—how those cancer-associated mutations might alter their enzymatic mechanism and affect nucleosome positioning. Here, missense mutations are of the highest interest, given the potential for gain-of-function and/or dominant-negative mutations.

Insight into how a mutation might create a gain-of-function/hyperactive remodeler enzyme derives from genetic and biochemical studies using yeast orthologs and through structural studies.

 © 2020 Elsevier Inc.

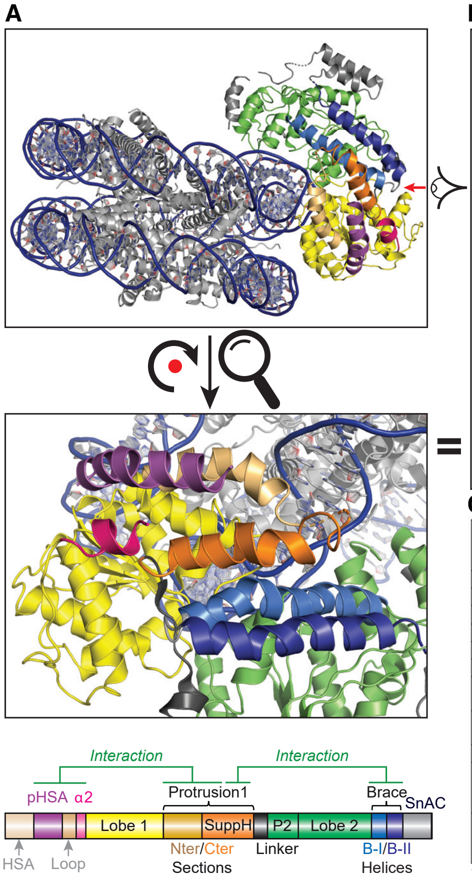

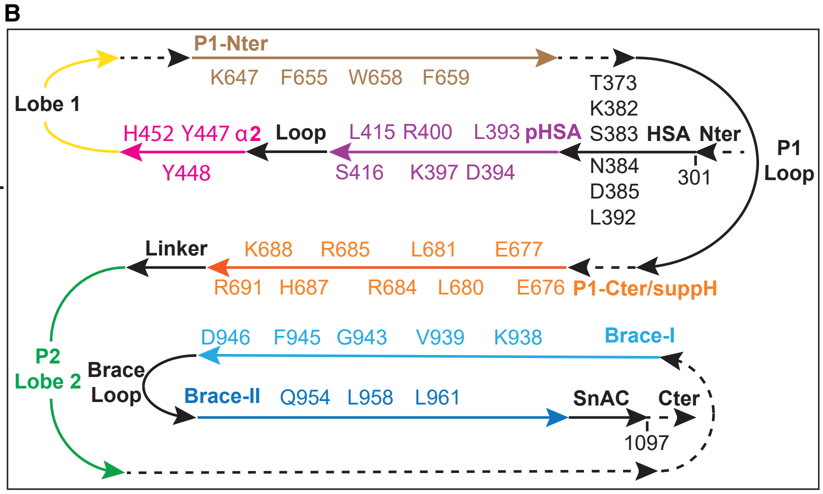

C

BRG1 Residue	Number of Samples & Mutations*	Tissue Distribution	Histology Subtype	Sth1 Residue & Mutation	
M527	1I	Large Intestine	AC	K382I	HSA
E533	1D	Kidney	ccRCC	N384D	
K546	3Δ	Liver, Stomach, H&L	Carcinoma, Lymphoid Neoplasm	K397Δ	pHSA
R549	1C/1L	Cervix / Soft Tissue	Squamous Cell Carcinoma / Sarcoma	R400C	
T565	2M	Large Intestine	AC	S416M	
H736	1R/1Y	Lung / Skin	AC / Carcinoma	H452Y	α2
R966	7W/1Q	Large Intestine, Ovary, Esophagus / H&L	AC, Serous Carcinoma / Lymphoid Neoplasm	R684W	suppH
R967	3H	Breast, Stomach, Urinary Tract	Carcinoma, Transitional Cell Carcinoma	R685H	
H969	2Y/1N	Skin / Lung	Squamous Cell Carcinoma / AC	H687Y	
G1232	17S/2D/3V/3C	Pancreas, Lung, CNS, Kidney, H&L, Stomach, Testis, Endometrium	AC, Carcinoma, ccRCC, Glioma, Lymphoid Neoplasm	G943S	Brace
R1243	22W/1L/1G/1P/1Q	Biliary Tract, Ovary, Pancreas, Stomach, H&L, CNS, Large Intestine	AC, Carcinoma, Glioma, Lymphoid Neoplasm	Q954W	

*: From COSMIC, the catalogue of somatic mutations in cancer; H&L: Haematopoietic & Lymphoid
CNS: Central Nervous System; AC: Adenocarcinoma; ccRCC: clear cell Renal Cell Carcinoma

Figure 1. An Integrative Regulatory Hub within the ATPase/Translocase of SWI/SNF Remodelers

(A) Top: structure of the Snf2 ATPase/DNA-translocase (residues 666–1,400) bound to nucleosome (PDB: 5X0Y) (Liu et al., 2017). As Sth1 is highly similar to Snf2 over this region, it serves as a structural model. Middle: magnification and rotation of the top panel, to reorient and focus on the structural hub, viewed from the perspective of the schematic eye. The red arrow (top panel) and red dot (below the top panel) depict the axis of rotation, and the black arrow the direction of rotation. The helices of the integrative regulatory hub (purple, post-HSA; fuchsia, α2; beige, protrusion 1 N-terminal section; orange, protrusion 1 C-terminal section (SuppH); light blue, brace-I; dark blue, brace-II) flank the ATPase/DNA-translocase lobes (yellow, lobe 1; green, protrusion 2 and lobe 2). Bottom: linear schematic of the Snf2/Sth1 ATPase/DNA translocase subunit, depicting the conserved regulatory regions and their interactions (green connectors). This schematic includes the HSA domain, which forms a long helix that binds ARPs (Schubert et al., 2013), but the HSA is not present in the Snf2 structure, above.
(B) Schematic representation of the hub, depicting all the residues investigated in this study, with each domain color-coded. Arrows depict domain direction (Nter-to-Cter), whereas dashed lines depict physical connectors/linkers between domains.
(C) Table listing 11 human BRG1 residues mutated in cancer, with the number of samples and mutations, tissue distribution, and histology subtype (COSMIC database) (Tate et al., 2019) and their corresponding residue in Sth1. Domains to which these mutations map depicted on the right.

Chromatin remodelers are molecular machines that harbor an enzymatic subunit with an ATPase domain that includes two RecA-like lobes separated by a DNA-binding cleft (Bartholomew, 2014; Clapier et al., 2017; Zhou et al., 2016). These two lobes perform ATP-dependent DNA translocation through an inchworm-like conformational cycle involving each of the two linked lobes moving one base along the DNA backbone during each ATP binding and hydrolysis cycle (Clapier et al., 2017; Velankar et al., 1999). In the context of a nucleosome, the two lobes bind two helical turns from the dyad (Figure 1A) (Saha et al., 2005; Schwanbeck et al., 2004; Zofall et al., 2006), and from that location, "pump" DNA around the nucleosome, causing nucleosome repositioning/sliding or ejection (Clapier et al., 2017). These two RecA-like lobes are flanked/linked by a set of helical SWI/SNF family-specific domains that regulate the lobes: the post-HSA and α2 domains (flanking lobe 1), the protrusion 1 domain (which includes an N-terminal helix and the C-terminal SuppH helix) located between the two lobes, and the brace domain (flanking lobe 2) (Flaus et al., 2006; Szerlong et al., 2008; Xia et al., 2016). Importantly, these domains fold together to reside at the physical junction between the RecA-like lobes (Liu et al., 2017), adjacent to the HSA domain C terminus, to form what we term a "structural hub" (Figures 1A and 1B). We find this structural hub conserved within a series of recent structural studies involving remodeler-nucleosome complexes (see Discussion) (Baker et al., 2020; Han et al., 2020; He et al., 2020; Liu et al., 2017; Wagner et al., 2020; Ye et al., 2019). A striking feature of the hub is the placement of the SuppH helix at its

central position. Notably, in the yeast ortholog of BRG1, termed Sth1, the post-HSA and protrusion 1 domains are known to regulate DNA translocation efficiency, and certain mutations in these domains create gain-of-function Sth1 proteins with high nucleosome sliding and ejection activity (Clapier et al., 2016).

Here, we hypothesized that this five-domain integrative hub regulates DNA translocation efficiency of the RecA-like lobes—and cancer-associated missense mutations in this hub might dysregulate by either hypo- or hyper-activation. To test, we conducted a genetic and biochemical examination of 25 alanine scanning mutations and 11 cancer-associated missense mutations using the closely related yeast Sth1 ortholog. Yeast RSC and recombinant Sth1 have been extensively studied and function as a paradigm to investigate the mechanism and regulation of SWI/SNF remodelers *in vitro* and *in vivo*. Remarkably, our results reveal two distinct categories of cancer-associated mutations: loss-of-function mutations that greatly impair DNA translocation and a novel category of gain-of-function mutations that upregulate DNA translocation efficiency and nucleosome remodeling *in vitro* and increase chromatin accessibility *in vivo*. Notably, each category maps to a distinct structural location within the integrative regulatory hub. This structure-function relationship is also strongly supported by a complementary and systematic alanine scanning approach.

Taken together, our results identify a new category of gain-of-function cancer-associated missense mutations and define a conserved SWI/SNF-family integrative structural hub with two distinct regions: one that regulates remodeler efficiency and another required for implementing remodeling—via the coupling of ATP hydrolysis into DNA translocation. Moreover, our results allow one to predict the consequences of a disease-associated missense mutation based on its location within the integrative regulatory hub of the SWI/SNF ATPase-DNA translocase motor subunit.

RESULTS

A System to Characterize the Sth1 Structural Hub and Gain-of-Function Mutations

To understand how the Sth1 structural hub regulates DNA translocation, and to determine whether certain cancer-associated missense mutations in *BRG1* create gain-of-function nucleosome remodeling activities, we introduced into STH1 25 alanine substitutions, and 11 cancer-associated missense mutations (chosen using the Catalogue of Somatic Mutations in Cancer [COSMIC]) (Tate et al., 2019) (Figures 1B and 1C). All residues targeted for mutation are either identical or highly similar between human BRG1 and yeast Sth1.

For each mutation, we expressed recombinant Sth1 protein in bacteria and purified proteins using affinity purifications and size-exclusion chromatography (see STAR Methods). Each Sth1 derivative is fused to the TetR protein, which enables the assessment of DNA translocation via a supercoiling assay, in which DNA translocation along the helical backbone confers, and is proportional to, increased supercoiling (see Figure S1 for diagram of assay). For each mutant Sth1 derivative, ATPase activity/value (termed *Av*) and DNA translocation efficiency (coupling value [*Cv*], expressed as supercoiling/ATP hydrolyzed) were measured and normalized to wild-type (WT) Sth1 (thus, *A*v and *C*v values for WT are 1.0). To address chromatin remodeling, we assessed mononucleosome sliding (200 bp 601 sequence, recombinant octamers) from a center-to-end position, but utilized conditions such that WT Sth1 only moves ∼10% of the nucleosomes, so increases in activity from gain-of-function derivatives can be easily visualized. We also monitored nucleosome ejection using a nucleosomal array formed on a closed circular plasmid (3.2 kb), using a 2D gel assay in which plasmid topoisomers can be clearly distinguished, and the loss of each nucleosome confers the successive loss of one supercoil. To investigate the impact of mutations *in vivo*, we conducted genetic and genomics experiments. For genetic assessments, we introduced these mutations into a plasmid-borne copy of *STH1* to test for *sth1*Δ complementation, or dominant-lethal growth inhibition of a WT strain (*STH1*$^{+}$). Finally, to determine their impact on genome-wide accessibility, we tested a subset (nine) of these mutations for chromatin openness by using a modified version of the transposase-accessible chromatin with high-throughput sequencing (ATAC-seq) approach.

Alanine Scanning Mutations Define Domain Roles and the Protrusion 1 SuppH Helix Interface

First, to conduct a systematic and nearly saturating analysis of the structural hub of Sth1, we performed alanine scanning—involving the substitution of 25 residues within the hub with an alanine residue (16 representative mutants depicted in Figure 2 [color-coded by domain]; remaining 9 mutants in Figure S2). Interestingly, 16 of these 25 alanine substitutions increased DNA translocation. These mutants generally displayed lower ATPase activity than WT, which was counterbalanced by a larger increase in coupling, resulting in increased DNA translocation. These 16 mutants all retained nucleosome sliding activity at levels that were comparable to or higher than WT, consistent with their impact on DNA translocation. Furthermore, five of the 16 additionally demonstrated increased nucleosome ejection (Y447A, W658A, F659A, R685A, and L393A) discussed below. Genetically, 15 of 16 complemented *sth1*Δ (W658A excepted) and 14 of 16 were not dominant lethal (W658A and F659A excepted) (Figures 2A, 2B, and S2A). Remarkably, these 16 residues all map to a distinct portion of the structural hub: the post-HSA domain (L393A, D394A, K397A, R400A, L415A, and S416A), the α2 helix (Y447A, Y448A, and H452A), the protrusion 1 N-terminal helix (K647A, F655A, and F659A), the side of SuppH helix (R685A and K688A) facing the post-HSA domain, and the extreme N terminus of brace helix 1 and in close proximity to SuppH (K938A). Based on this clustering, we will hereafter refer to this distinct region of the structural hub as "region 1."

As previewed above, five region 1 mutations displayed increased nucleosome ejection. First, Y447A (α2 helix) exhibited moderately higher ATPase activity and moderate coupling, whereas W658A (N-terminal helix, protrusion 1) retained normal coupling while greatly increasing ATPase activity (Figures 2A and S2A). Notably, each displayed moderate or high nucleosome ejection *in vitro* in the closed circular array system, respectively. Mutations L393A (post-HSA), F659A (N-terminal helix, protrusion 1), and R685A (SuppH helix, facing the post-HSA) all increased nucleosome ejection and displayed near-WT ATPase activity with high coupling (Figures 2A and 2B). Thus,

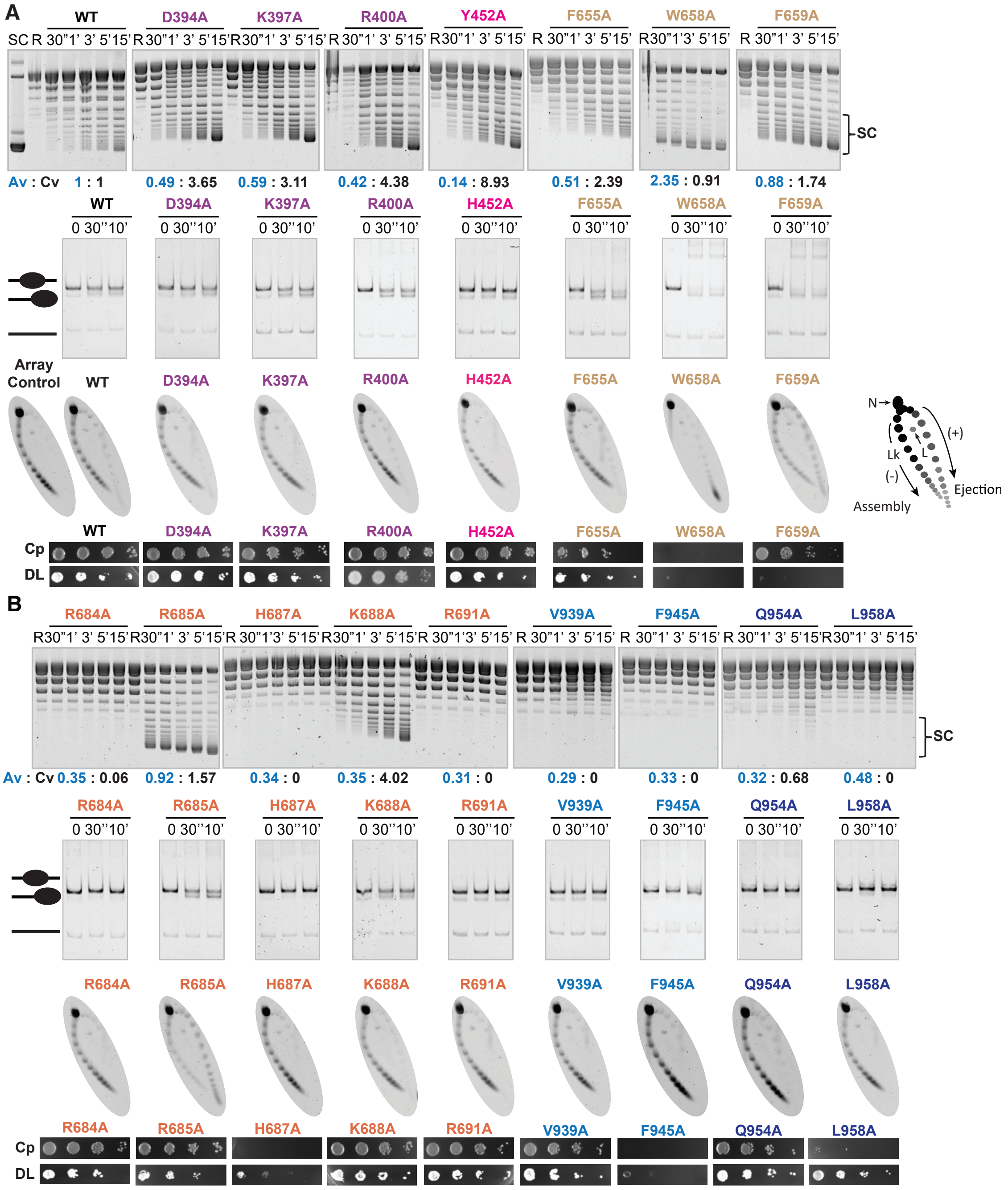

Figure 2. Alanine Substitutions Either Enhance or Abolish DNA Translocation, Coupling, and Nucleosome Sliding, Revealing Two Distinct Functional Regions within the Hub

(A) Comparative impact of alanine substitutions within region 1. First row: comparative Tet-tethered DNA translocation activity measured by the accumulation of plasmid supercoiled (SC) topoisomers. Sth1 is tethered to the TetO containing plasmid via fusion to the DNA-binding domain of TetR (forming TetR-Sth1301–1097). Translocation along the DNA backbone creates positive supercoils in front of the translocase, and negative supercoils behind. E. coli Topoisomerase I (present in the reaction) relaxes only negative supercoils; thus, translocation yields one positive supercoil/10 bp translocation. The plasmid-stimulated ATPase activity/value (blue text, Av) and coupling value (black text, Cv, expressed as supercoiling/ATP hydrolyzed) and normalized to WT Sth1 (thus, Av and Cv values for WT Sth1 are 1.0). SC, highly supercoiled topoisomers; R, relaxed plasmid. Second row: comparative sliding of mononucleosomes (601 positioning DNA, 200 bp). Third row: comparative nucleosome ejection in a closed circular array format. At right: schematic of the principle of the nucleosome array ejection assay, with SC plasmid (topoisomer) distribution revealed by a 2D gel. Lk, linking number; N, nicked; L, linear. In sliding and ejection assays, the enzyme:nucleosome molar ratio is 1:2, and representative gels from multiple experiments are shown. Fourth row: comparative genetic assessment (complementation [Cp] and dominant-lethality [DL]) conferred by expression of particular Sth1 derivatives. Ability to complement assessed by expressing transformed Sth1 derivatives (regulated by WT STH1

(legend continued on next page)

improved ejection is observed only when both parameters (ATPase and coupling) are near WT or well above WT, and one parameter must be well above WT—a combination reminiscent of L392P (Clapier et al., 2016), which also displayed high nucleosome ejection. Furthermore, the mutations that greatly upregulate nucleosome ejection (e.g., W658A, F659A, and L392P) are among those that lack *sth1*Δ complementation or display dominant lethality. Taken together, region 1 negatively regulates both ATPase and coupling, and mutations in this region typically improve DNA translocation and remodeling.

In contrast, 9 of these 25 alanine substitutions led to uncoupling, signified by an absence of DNA translocation or nucleosome remodeling (sliding or ejection) while retaining moderate ATPase activity (Figures 2B and S2A). Remarkably, all 9 residues in this category mapped to domains within the "second half" of the structural hub, hereafter named "region 2": the brace helices with V939A, F945A, D946A, Q954A, L958A, and L961A, and remarkably, the side of SuppH helix of protrusion 1 facing the brace with R684A, H687A, and R691A.

Curiously, certain mutations (R684A, R691A, V939A, Q954A, and L961A) that lacked DNA translocation were able to partially complement the loss of *STH1* in our genetic assays; an unexpected result, because DNA translocation is considered a fundamental property. Here, we reasoned that the presence of the ARP module (Arp7, Arp9, and Rtt102), which is known to increase coupling (Clapier et al., 2016), might partially rescue DNA translocation of these Sth1 mutants *in vitro* and *in vivo* and thus resolve the discrepancy. To test, we examined R684A and Q954A mutations in the presence of the ARP module (Sth1, ARP, Rtt102 [SAR]), as well as H687A and F945A, two mutations that displayed the expected biochemical and genetic results (Figure S2B). Consistent with this explanation, the addition of the ARP module partially restored DNA translocation with R684A and Q954A mutations, and moderately restored nucleosome sliding with SAR-R684A (more subtly with SAR-Q954). In counter distinction, ARP module addition to mutants with expected behaviors (H687A and F945A) did not improve DNA translocation (Figure S2B). Thus, ARP module restoration *in vitro* helps reconcile discrepancies between biochemical and the genetic results of Sth1 mutants.

Taken together, our alanine scanning results defined two functional regions of Sth1: region 1 regulates coupling and ATPase activity (mutations confer upregulation), whereas region 2 implements coupling (mutations prevent coupling). Notably, the SuppH helix resides at the interface of these regions, and mutations on SuppH that face region 1 upregulate Sth1, whereas those that face region 2 inactivate Sth1 by preventing coupling.

Mutations that Suppress *arp*Δ Mutations Increase Coupling and DNA Translocation

Previous genetic work revealed 10 mutations within *STH1*, termed *mra* mutants that modify the requirement for ARPs, which restore viability to *arp*Δ mutants (Szerlong et al., 2008). These 10 *mra/sth1* alleles map to two specific locations: to the transition between the HSA and post-HSA domains (T373P, K382N, N384K, D385Y, and L392V) or to protrusion 1 (E676Q, L680M, L680V, L681F, and K688T), and thus all map to the structural hub. Prior work characterized three of these *mra* mutations, which all improved DNA translocation (Clapier et al., 2016). Now, access to the structure, along with the alanine scanning results designating two functional regions, motivated our characterization of the remaining seven *mra* mutants as well, to test functional consistency with the other mutations in region 1. Here, all 10 *mra* mutations complemented *sth1*Δ (Figure 3A). Notably, all 10 improved DNA translocation, and the vast majority improved coupling—explaining how the *STH1* mutations can functionally compensate for the lack of the ARP module (Figure 3A). Furthermore, all of these *sth1/mra* alleles map to region 1 of the hub, consistent with these mutations upregulating coupling and DNA translocation.

We then tested all five isolated dominant lethal *STH1* mutations isolated previously (Clapier et al., 2016). In counter distinction to the *mra* alleles, dominant lethal mutations in Sth1 (e.g., S383P, L392P, Δ[D385-L392], E677G, and L681S) greatly increased ATPase activity and DNA translocation, without improving coupling (Figure 3B), reminiscent of the dominant lethal W658A mutation in region 1 from our alanine scanning. Of note, the dominant lethal mutations/deletion that map to the transition between the HSA and post-HSA domain led to a much more pronounced increase in DNA translocation than those that map to protrusion 1. Taken together, the lethality observed upon the loss of ARPs (which lowers DNA translocation) can be suppressed by mutations in region 1 of the hub that moderately increase DNA translocation, most often through improving coupling; whereas mutations that greatly improve DNA translocation by increasing ATPase activity confer lethality.

Gain-of-Function Cancer-Associated Missense Mutations Improve DNA Translocation Efficiency and Co-localize in Region 1

Prior work focused on cancer-associated mutations that map to the BRG1 RecA-like lobes, preventing remodeling (Bultman et al., 2005; Dykhuizen et al., 2013; Hodges et al., 2018). In contrast, our eleven cancer-associated mutations do not map to the RecA-like lobes themselves; they exclusively map to the structural hub, and involve the transition between the HSA and post-HSA domains (K382I and N384D), the post-HSA domain (K397Δ, R400C and S416M), the α2 helix (H452Y), the SuppH helix (R684W, R685H, and H687Y) and the braces (G943S and Q954W) (Figure 1C, color-coded by domain).

To understand their mechanistic impact, we tested all eleven cancer-associated mutations in all biochemical assays (Figure 4). Remarkably, the seven mutants that map to region 1, including the SuppH mutation that faces the post-HSA domain within region 1 (R685H), all conferred a moderate reduction in ATPase activity, while simultaneously increasing DNA translocation—and thus improve the coupling of ATP hydrolysis to DNA

promoter) in cells lacking genomic WT STH1. DL status assessed by expression using the methionine-regulated MET25 promoter, in the presence of WT STH1. Each panel displays a dilution series of yeast cells on plate media, testing growth ability. One representative of three replicates is shown. Mutations are color-coded according to the domains they belong to in the hub, as in Figure 1.
(B) Comparative impact of alanine substitutions in the SuppH helix and region 2. All assays performed and results depicted as in (A).

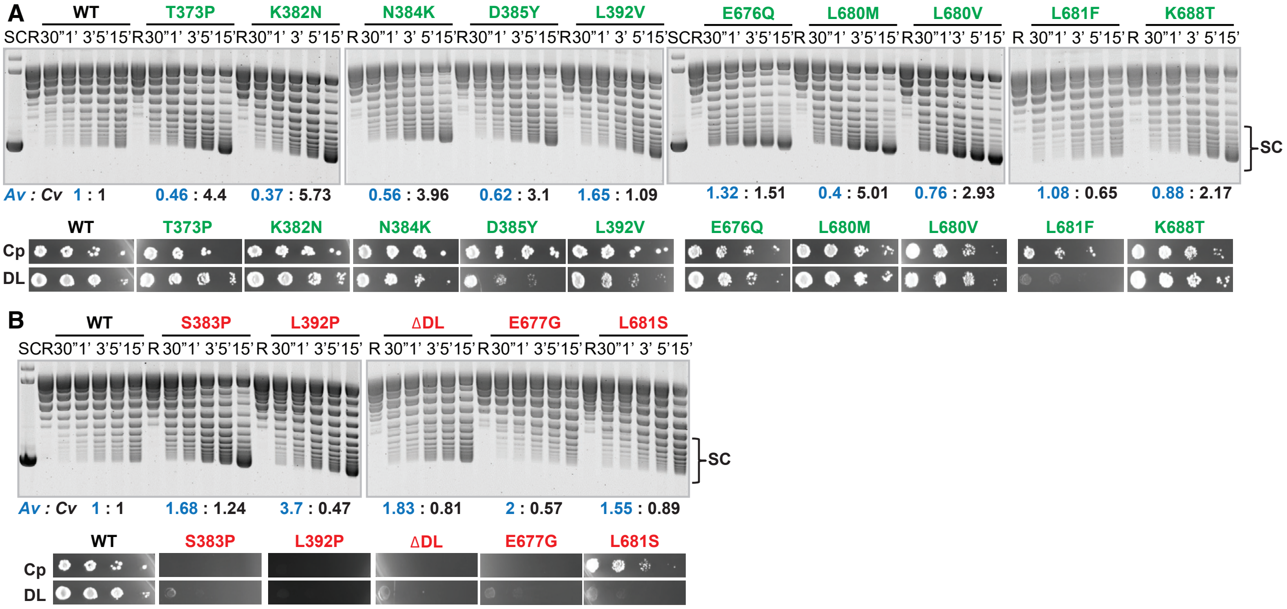

Figure 3. Mutations that Suppress *arp*Δ Mutations or Exhibit Dominant Lethality Map within Region 1

(A) Mutations that suppress *arp*Δ mutations (*mra* mutants) increase coupling and DNA translocation, complement the loss of WT Sth1, and map to region 1. First row: comparative ATPase and DNA translocation assays performed using Sth1 derivatives harboring the 10 *arp*Δ-suppressing *mra* mutations (green), five clustered in the HSA, and five clustered in the SuppH of protrusion 1 (Szerlong et al., 2008). Second row: comparative genetic assessment as in Figure 2A.

(B) Mutations that confer dominant lethality increase ATPase activity and DNA translocation fail to complement the loss of WT Sth1 and map to region 1. First row: comparative ATPase and DNA translocation assays performed using the five DL Sth1 mutants (red) located in the HSA and the SuppH of protrusion 1. Second row: comparative genetic assessment as in Figure 2A.

translocation (Figure 4A, and mutants H452Y and R685H in Figure 4B). Furthermore, all seven displayed increased nucleosome sliding but did not display increased nucleosome ejection either in a mononucleosome format or in a closed circular array format. Moreover, six of these seven mutations complemented *sth1*Δ (K397Δ excepted) and none were dominant lethal, suggesting that this extent of increase in nucleosome sliding by Sth1 is compatible with viability. Taken together, the seven mutations that map to region 1 of the structural hub are all gain-of-function cancer-associated missense mutations that increase DNA translocation and nucleosome sliding, but not nucleosome ejection.

Loss-of-Function Cancer-Associated Missense Mutations Co-localize Spatially and Greatly Reduce DNA Translocation Efficiency

Four cancer-associated mutations mapped to region 2 of the structural hub; two (G943S and Q954W) mapped to the brace helices and two (R684W and H687Y) mapped to the side of the SuppH helix that faces the brace helices in region 2 (Figures 1B and 1C). Each of these mutations eliminated DNA translocation while retaining DNA-dependent ATPase activity, generating fully uncoupled ATPases that were unable to perform nucleosome sliding or ejection, failed to complement *sth1*Δ, and were not dominant lethal (Figure 4B). Thus, we define a category of loss-of-function cancer-associated missense mutations, within region 2, which confer uncoupling.

Notably, our alanine scanning overlapped with many of the residues bearing cancer-associated missense mutations; here, K397A, R400A, H452A, R684A, R685A, H687A, and Q954A pair with K397Δ, R400C, H452Y, R684W, R685H, H687Y, and Q954W, respectively. Importantly, the genetic and biochemical results for each pair were consistent (allowing for modest variation from the alanine substitution), additionally validating the function of each residue. Their overall similarity also supports the notion that alanine scanning has predictive value as a proxy for the functional consequences of disease-associated missense mutations. Taken together, cancer-associated missense mutations in Sth1 can be classified into one of two categories depending on their locations: gain-of-function mutations located in region 1 that improve coupling or loss-of-function mutations located in region 2 that lose coupling. Interestingly, the highly conserved protrusion 1 SuppH helix resides at the interface of these two regions, harbors mutations belonging to each category, and the impact of those mutations is predicted by whether they face region 1 or region 2.

Gain-of-Function *STH1* Mutations Open Chromatin

Our collective results strongly suggest that STH1 hub mutations partition into three categories, relative to WT Sth1: (1) loss-of-function non-complementing mutations (low-moderate ATPase, no coupling), (2) gain-of-function yet viable mutations (low-moderate ATPase, increased coupling), and (3) gain-of-function inviable/dominant-lethal mutations (increased ATPase, moderate-WT coupling). In keeping, graphical representation of the *Av* and *Cv* values for this spectrum of *STH1* hub mutations colocalizes the members of each category

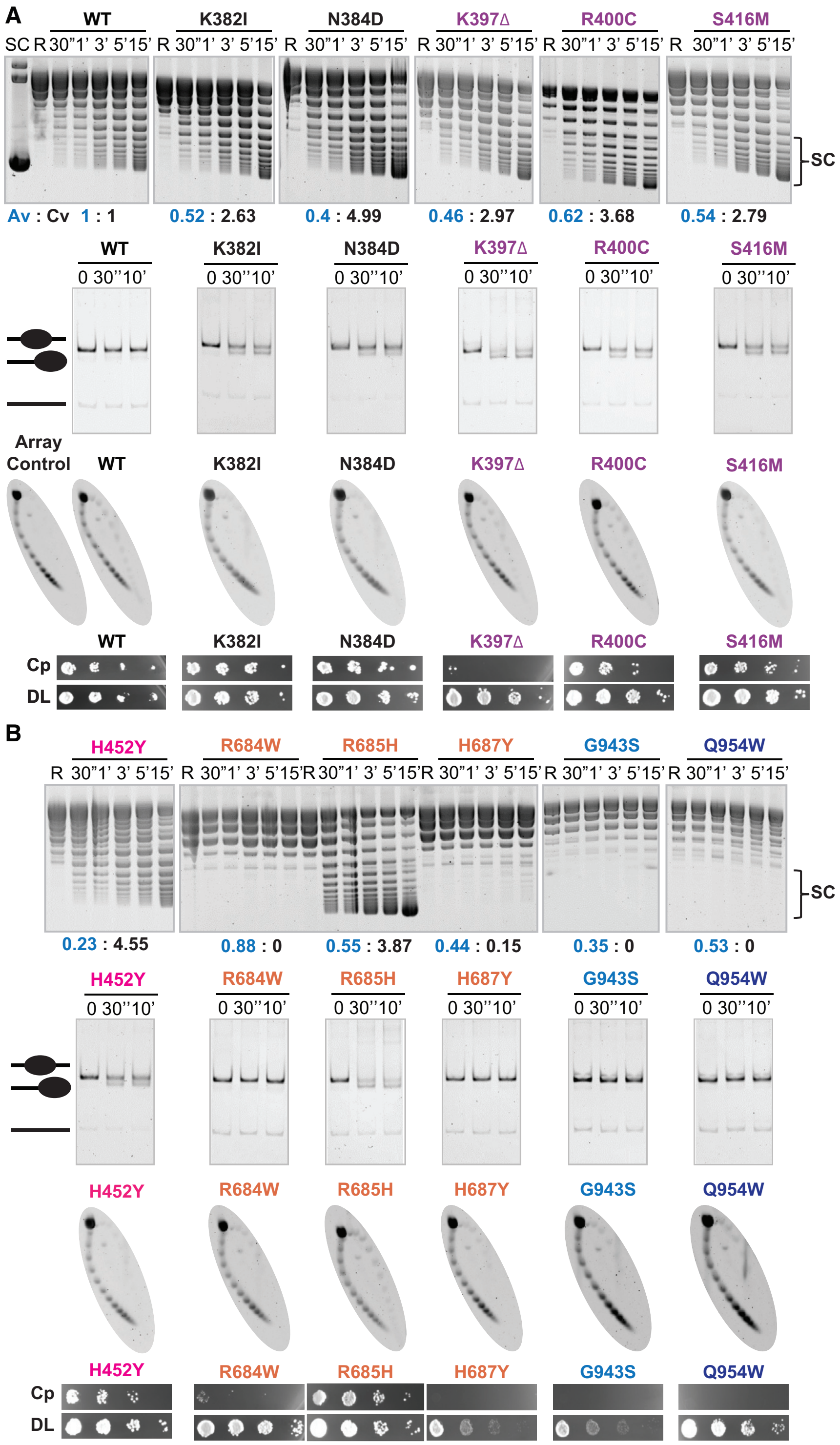

Figure 4. Cancer-Associated Missense Mutations Either Enhance or Abolish DNA Translocation, Coupling, and Nucleosome Sliding, Confirming Two Distinct Functional Regions within the Hub

(A) Cancer-associated missense mutations within regulatory region 1 enhance DNA translocation coupling and nucleosome sliding. All assays performed and results depicted as in Figure 2A.

(B) Cancer-associated missense mutations in the α2 helix, SuppH helix, and region 2 reveal the interface between regulatory region 1 and the implementation region 2. All assays performed and results depicted as in Figure 2A.

(Figure S3). Notably, all the viable cancer-associated mutations reside in category 2, which mapped to region 1, and increased nucleosome sliding *in vitro*.

To visualize the possible impact of gain-of-function human cancer-associated missense mutations on chromatin organization *in vivo*, in comparison to the two other mutation categories, we performed ATAC-seq experiments in *S. cerevisiae*, which provide accessibility profiles that are largely the inverse of nucleosomal occupancy profiles (Figure S4A). Here, we reasoned that improvement in RSC nucleosome sliding would confer greater chromatin openness, perhaps genome-wide, due to the presence of RSC at most genes. To test, we examined mutants from each of the three categories of mutations described above: (1) three loss-of-function mutants (R684W, H687Y, and Q954W), (2) two gain-of-function cancer-associated mutants (R400C and R685H) and the *mra* mutation N384K, and (3) three dominant-lethal mutants (L392P, W658A, and L681F).

First, we modified the existing ATAC-seq protocol (Buenrostro et al., 2015) by including a crosslinking step and then optimized transposase 5 (Tn5) levels in a WT strain, which provided very clear Tn5 integration at known nucleosome-deficient regions—thus providing a new method for ATAC-seq profiling in yeast that utilizes crosslinked chromatin. Sth1 mutant derivatives (and WT Sth1) were then expressed with a regulatable (*MET25*) promoter for a length of time (1.5 h induction, determined via pilot experiments) that ensured moderate expression and impact, but prior to the expressed dominant-lethal mutations negatively impacting growth. To determine whether the expressed Sth1 mutant proteins assembled into RSC, we examined FLAG-immunoprecipitates from whole cell lysates in a native protein gel by immunoblotting, which quantified the location of FLAG-tagged Sth1—either as a faster migrating free subunit or as a part of the slower migrating RSC complex. Here, the large majority of

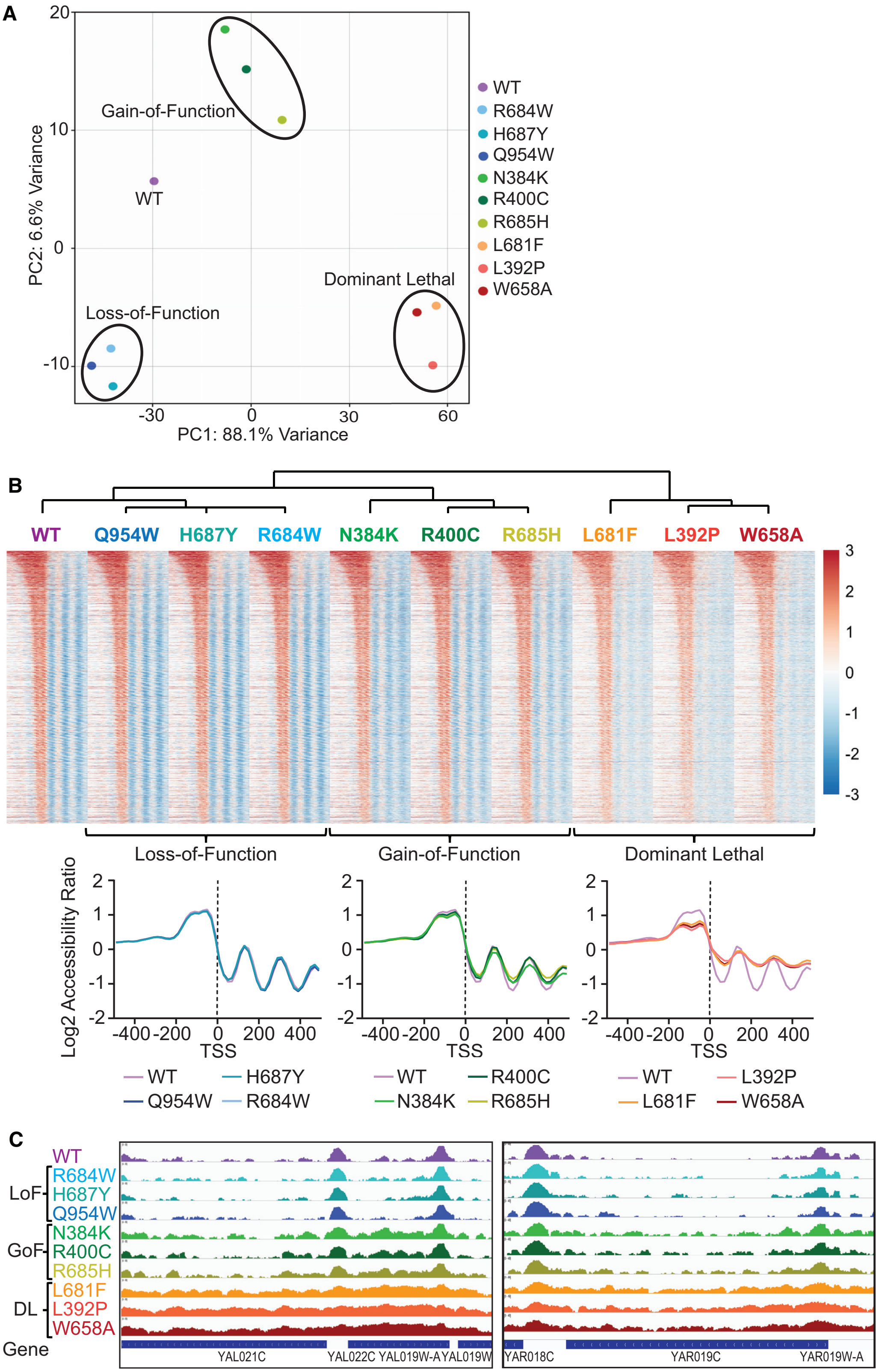

(legend on next page)

Sth1 protein (between 72% and 98%) with each mutant derivative assembled into RSC, compared to ~91% with WT STH1 (Figure S4B). Furthermore, those mutants that assembled less well than WT did not correlate with greater chromatin openness, suggesting that the category/activity of the Sth1 mutant in RSC, and not its action as a free subunit, underlies chromatin openness.

Overall, the ATAC-seq results had two main features. First, the three tested mutants within each of the three categories defined above (loss-of-function, gain-of-function, and dominant lethal) clustered together and thus impacted chromatin openness in a very similar manner (Figures 5A, principal-component analysis [PCA] plot, and S4C, dendrogram). Second, when comparing across the nine mutants, we observed a gradient of effect in openness (heatmap and accessibility profiles) (Figure 5B), which could be grouped into three categories: category 1, no change in chromatin openness (compared to WT) with the loss-of-function cancer-associated mutations (R684W, H687Y, and Q954W), an expected result, as the mutations were covered by a WT *STH1* allele (see STAR Methods); category 2, a moderate increase in openness with the viable gain-of-function mutations (*mra* N384K and gain-of-function cancer-associated mutations R400C and R685H); and category 3, a major genome-wide increase in openness with dominant lethal mutations (L392P, *mra* L681F, and W658A). To illustrate this gradient of impact, we provide mean chromatin accessibility profile maps (Figure 5B, lower panels) and representative snapshots from the genome browser (Figures 5C and S5, gallery). Interestingly, the three separate clusters identified by PCA and heatmap analysis of the genomic data mirror the three categories defined by the *A*v/*C*v scatterplot described above (Figure S3) and the nucleosome remodeling results. For example, the moderate alteration in chromatin openness/nucleosome positioning observed with the gain-of-function cancer-associated mutations aligns with their increased nucleosome sliding *in vitro*, whereas the extensive impact on chromatin openness with the dominant lethal mutations aligns with their improved sliding and nucleosome ejection activity *in vitro*. Regarding cancer, these results obtained from the yeast system strongly suggest that the gain-of-function cancer-associated mutations tested will precociously slide nucleosomes, open chromatin, and confer promiscuous gene expression in human tumors.

DISCUSSION

A Structural Hub in Sth1 Functions as an Integrative Regulatory Hub for Chromatin Remodeling

Here, we utilized the tools available in the yeast Sth1/RSC system to determine whether certain cancer-associated mutations in BRG1/BAF complex confer gain-of-function properties, and if so, to address the mechanism. The structure-regulatory logic of SNF2-family ATPases appears to involve an "integrative regulatory hub" that influences the rate of ATP turnover and the efficiency of DNA translocation by the RecA-like lobes. A summary table of all 51 hub mutations investigated, along with their behaviors in all assays, is presented in Table S1. Our work provides several lines of evidence that this hub consists of two structurally and functionally distinct regions: region 1 domains consist of the transition between the HSA and post-HSA domains, the post-HSA, the α2-helix, the N-terminal helix of protrusion 1, and the SuppH residues facing these domains. In counter distinction, region 2 domains include the brace helices and the SuppH helix residues interacting with the brace. Region 1 regulates coupling; because mutations in this region upregulate coupling, region 1 acts to restrain coupling (Figures 6A and 6B). Here, we reason that activators and/or histone modifications might interact with region 1 (or the ARPs, which bind to the HSA) to upregulate coupling and thus improve sliding or enable ejection in the course of gene regulation. In contrast, region 2 is required for the implementation of coupling, because mutations in this region generally eliminate DNA translocation and sliding, while maintaining low ATPase activity.

SuppH of Protrusion 1 Bridges the Regulatory and Implementation Regions of the Hub

Interestingly, the protrusion 1 SuppH helix resides at the interface of region 1 and region 2; each side of the SuppH helix conducts functional roles that align with the region it faces (Figures 6A and 6B). Thus, SuppH appears well-positioned to integrate (perhaps via a conformational change) regulatory inputs imposed on region 1 and to relay them to region 2. The SuppH helix itself is structurally conserved from yeast to human and present in the ATPases of all remodeler families (Flaus et al., 2006; Xia et al., 2016). However, only the latter portion of SuppH is conserved among all remodeler families, whereas the beginning of SuppH is extended in length in SWI/SNF-family remodelers.

Figure 5. Gain-of-Function Cancer-Associated Mutations and Dominant Lethal Mutations Open Chromatin Moderately and Extensively, Respectively

(A) Principle component analysis of ATAC-seq data partitions Sth1 hub mutants into three clusters. A modified ATAC-seq protocol (employing crosslinking) was applied to a subset of hub-localized *sth1* mutants from three categories: (1) loss-of-function cancer-associated mutants (Q954W, H687Y, and R684W) from region 2, (2) gain-of-function *mra* (N384K) or cancer-associated (R400C and R685H) mutants from region 1, and (3) dominant lethal mutants (L681F, L392P, and W658A) from region 1. All Sth1 derivatives were expressed using a *MET25* inducible promoter. The mean log2 fold ratio of signal over background was collected in a 1-kb window (−500 to +500 bp) of protein-coding gene TSS, and the first two principal components were plotted for the top 1,000 variant genes. Phenotypic categories are circled for emphasis.

(B) Genome-wide impact of Sth1 hub mutations on chromatin structure at promoters. Top: a hierarchical clustering tree/dendrogram derived from the mean accessibility heatmap over 1 kb of all TSS (Figure S4C) shows that the *sth1* mutants cluster into three categories. Middle: heatmaps of log2 fold ratio of DNA accessibility signal over genomic background at all promoter TSS (1 kb window, −500 to +500 bp) arranged in descending order of promoter NDR length. The *sth1* mutants within each category appear similar, while the three categories display a progressive impact. Bottom: log2 mean profile of DNA accessibility ratio over 20 bp bins at all TSS (1-kb window, −500 to +500 bp) plotted to depict changes in accessibility between mutants from the three categories.

(C) Genome browser snapshots depicting the impact of Sth1 mutants on chromatin openness. Accessibility coverage (log2 RPM coverage) across two representative chromosomal regions is shown. All tracks are log scaled to the same y axis.

A

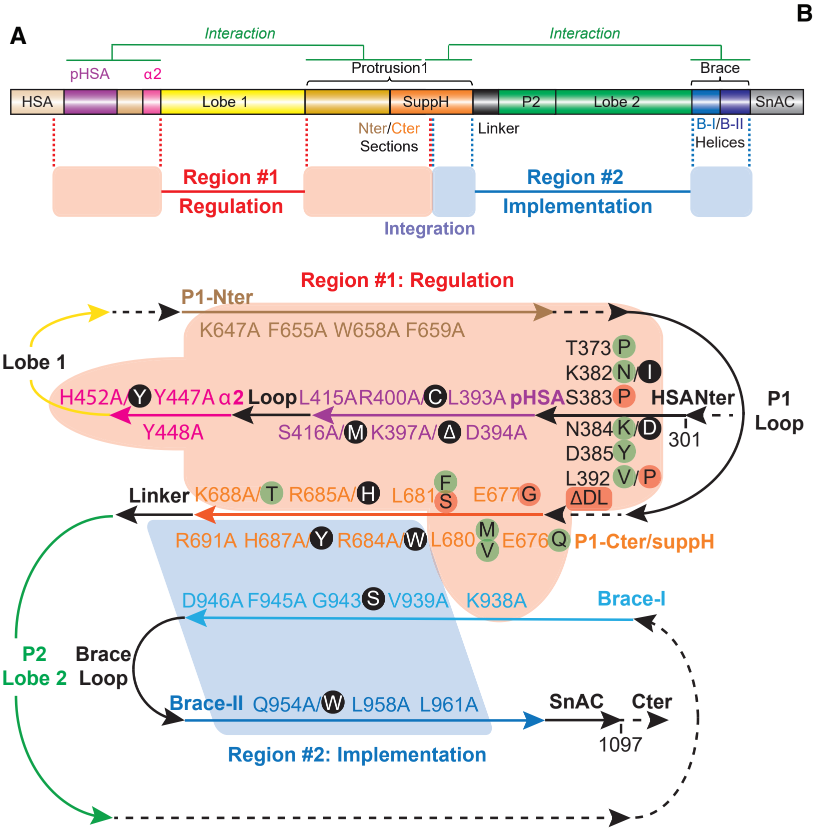

B

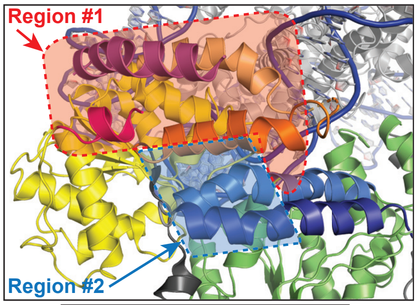

C

Structure		M.t.	Nucleosome				
		Snf2	S.c. Snf2	S.c. RSC		S.c. SWI/SNF	Human BAF
PDB ID		5HZR	5X0Y	6KW3	6TDA	6UXW	6LTJ
Hub Domains	pHSA	✓	✓	✓	✓	✓	✗
	α2	✓	✓	✓	✓	✓	✗
	P1 Nter	✓	✓	✓	✓	✓	✓ (Partial)
	P1 Cter (SuppH)	✓	✓	✓	✓	✓	✓ (Partial)
	Brace-I	✗	✓	✓	✓	✓	✗
	Brace-II	✗	✓	✓	✗	✓	✗
Hub Depiction in Figure		**6D1**	**6D2**	**6D3**	**6D4**	**6D5**	**6D6**
		N/A	**6E1**				N/A
		N/A		**6E2**			N/A

D

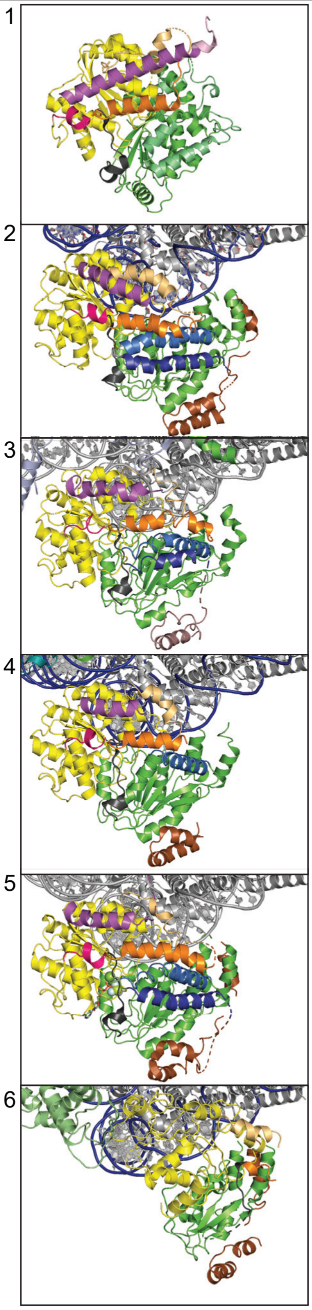

E

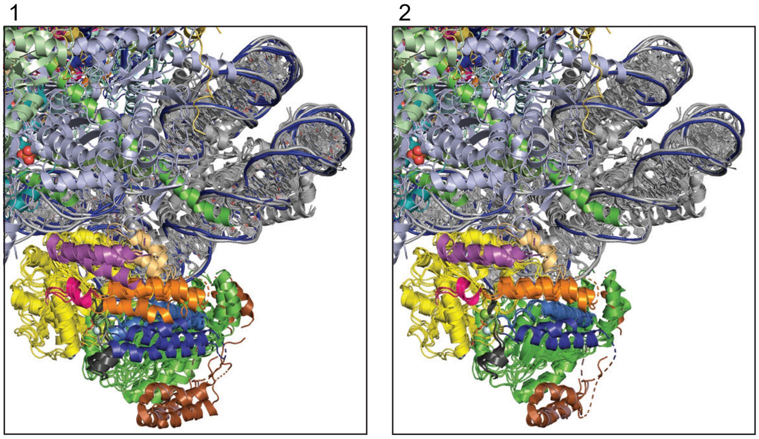

(legend on next page)

This SWI/SNF-specific extension harbors all but one (K688T) of the protrusion 1 *mra* mutations (which suppress *arp*Δ mutations and restore coupling), supporting a critical role for the extension in regulating coupling in SWI/SNF remodelers. Furthermore, the conserved portion of the SuppH helix in the ISWI ATPase appears to also play an interesting regulatory role: it binds directly to AutoN, a domain that negatively regulates the ATPase activity of ISWI (Yan et al., 2019). Notably, the sole *mra* mutation (K688T) in Sth1 that localizes to the conserved region of SuppH changes the amino acid to the threonine present in WT ISWI. Taken together, the SuppH domain, through extension or changes in amino acid composition, appears to specialize regulation during remodeler evolution. More specifically, we speculate that regulatory domains such as the post-HSA domain in SWI/SNF remodelers and the AutoN domain in ISWI have co-evolved with the SuppH helix to impart specialized regulation.

Gain-of-Function Cancer-Associated Mutations Co-locate and Upregulate Chromatin Remodeling

Here, we show that a particular category of cancer-associated missense mutations—those that co-locate in region 1 of the hub—upregulate coupling, moderately improve both DNA translocation and nucleosome sliding *in vitro*, but do not cause strong nucleosome ejection. They also complement a *sth1*Δ mutation and moderately promote chromatin openness *in vivo*—and are thus moderate gain-of-function mutations (Figure 4A). We note that *BRG1* gain-of-function mutations that map to region 1 are relatively rare compared to loss-of-function mutations, which map to many regions in BRG1 (including region 2), and are likely more easily acquired. Prior work characterized common cancer-associated and other missense mutations in *BRG1*, which localize to the surface of the RecA-like lobes, the ATP-binding pocket, or the braces, and impair chromatin remodeling *in vitro* or diminish DNA accessibility *in vivo*, and therefore are loss-of-function missense mutations (Bultman et al., 2005; Dykhuizen et al., 2013; Hodges et al., 2018). Importantly, all of the prior tested mutations map outside of region 1. Region 1 also contains all the *mra* mutations isolated via genetic suppression experiments to restore growth ability to strains lacking ARPs, among which 9 of the 10 are shown here to upregulate coupling (Figure 3A). Thus, *mra* mutations resemble cancer-associated missense mutations functionally. In contrast, because region 2 is required to implement coupling, cancer-associated missense mutations located in region 2 lack coupling, do not complement, and are thus loss-of-function mutations (Figure 4B). A third category of Sth1 mutations displays potent upregulation of ATPase activity while largely retaining coupling, improved nucleosome sliding—and the ability to eject nucleosomes at high frequency *in vitro*. *In vivo*, these mutants display dominant lethality and show a profound impact on chromatin openness/accessibility. Finally, we note that an upregulation of coupling has also been observed with one cancer-associated mutation (H1196Y) within the human CHD4 remodeler (Kovač et al., 2018), which maps to G937 in the brace-I helix of Sth1. Notably, the adjacent residue in Sth1, G938, when mutated to alanine (G938A) in our study, behaves as a gain-of-function mutation that improves coupling. Here, we speculate that these adjacent mutations in CHD4 and Sth1 both confer a brace conformation that promotes coupling and further support the notion that alteration of coupling may occur more broadly in cancers.

Taken together, our results strongly suggest that region 1-localized gain-of-function cancer-associated mutations in human BRG1 create a remodeler that may precociously slide nucleosomes and open chromatin to a moderate extent. This impact is distinct from the dominant lethal mutations, which are a highly dysregulated category of gain-of-function mutations that confer widespread nucleosome loss via ejection, a property that would likely impose cell arrest and/or death rather than growth/cancer. In contrast, the moderate dysregulation by cancer-associated mutations is predicted to be heterogeneous, creating patterns that can undergo iterative selection to arrive at an epigenome and transcriptome that is well adapted for growth and/or toleration of subsequent mutations along the path to cancer. This general mode of dysregulation can impact

Figure 6. The Domains Flanking the ATPase/DNA Translocase Lobes Form a Conserved Integrative Hub with a Regulatory Region 1 Where Cancer-Associated Gain-of-Function Mutations Map

(A) Top: linear schematic depicting the functional roles and physical interaction of Sth1 hub domains. The regions flanking the two RecA-like lobes associate structurally to form an integrative regulatory hub comprised of two distinct regions: region 1 is formed by the HSA, the post-HSA, and the Nter and parts of the Cter helices (including one side of SuppH) of protrusion 1, and is involved in the regulation of coupling (light red connected boxes); and region 2, formed by one side of SuppH helix of protrusion 1 and the braces, involved in the implementation of coupling (light blue connected boxes). Remarkably, the Cter helix of protrusion 1 (termed SuppH) appears to be split between region 1 and region 2, integrating the regulation and directing its proper implementation. Bottom: detailed schematic model of the Sth1 integrative regulatory hub. All domains and mutations investigated are depicted, revealing two functional regions: region 1 (light red shape) involved in DNA translocation/coupling regulation and harboring gain-of-function mutations (cancer-associated missense mutations [black dots] and alanine substitutions), the *mra* mutations (green dots), and the DL mutations (red dots), and region 2 (light blue shape) involved in the implementation of coupling and harboring loss-of-function mutations (cancer-associated missense mutations [(black dots] and particular alanine substitutions).

(B) Magnification of the integrative regulatory hub of Snf2 depicted as in Figure 1A, with the two revealed functional regions highlighted: region 1 (light red shape) and region 2 (light blue shape).

(C) Comparison of structural hub domains visible (green tick symbol) or not built in the structural model (red cross symbol) in the structures of various remodelers (structure names and PDB IDs mentioned) and their depiction in (D).

(D) Gallery of individual structural hubs from various remodelers aligned. (1) *Myceliophthora thermophila* Snf2 (PDB: 5HZR) (Xia et al., 2016). (2) *S. cerevisiae* Snf2 ATPase bound to a nucleosome (PDB: 5X0Y) (Liu et al., 2017). (3 and 4) *S. cerevisiae* RSC bound to a nucleosome (PDB: 6KW3 and 6TDA, respectively) (Wagner et al., 2020; Ye et al., 2019). (5): *S. cerevisiae* SWI/SNF bound to a nucleosome (PDB: 6UXW) (Han et al., 2020). (6) Human BAF bound to a nucleosome (PDB: 6LTJ) (He et al., 2020).

(E) Structural alignment of various remodeler-nucleosome complexes (oriented as in D) based on the alignment of their respective histone octamers highlighting the structural conservation of the different domains forming the hub. (1) Alignment of *S. cerevisiae* Snf2 ATPase (PDB: 5X0Y), RSC complex (PDB: 6KW3 and 6TDA), and SWI/SNF complex (PDB: 6UXW), each bound to a nucleosome. (2) As in (1) but without the Snf2 ATPase structure.

chromatin in any cell type, and therefore is not predicted to predispose to a particular type of cancer—consistent with the wide range of cancers linked to region 1 mutations (Figure 1C). Importantly, our results have predictive value regarding the likely consequences of a disease-associated missense mutation, based on its location within the integrative regulatory hub: if it maps to the regulatory region (region 1), it is likely to be a gain-of-function mutation characterized by increased coupling, whereas if it maps to the implementation region (region 2), it is likely to be a loss of function mutation that lacks coupling.

The Structural Hub Is Conserved in SWI/SNF-Family Remodeler-Nucleosome Structures

The recent structures of several SWI/SNF-family chromatin remodelers, either in a free form (Xia et al., 2016) or bound to a nucleosome (Han et al., 2020; He et al., 2020; Liu et al., 2017; Wagner et al., 2020; Ye et al., 2019), provided the opportunity to explore the structural conservation of the regulatory hub. First, we assessed the presence or absence of the domains forming the structural hub in the solved structures (Figure 6C) and depicted each individual hub separately (Figure 6D). We then performed a structural alignment based on the conserved histone octamers present in those structures using PyMOL (Schrödinger, 2019) (Figure 6E1). Here, the human BAF-nucleosome structure was not included, because the built structure had limited resolution and lacked the vast majority of the hub domains (Figures 6C and 6D6). Interestingly, the remodeler-nucleosome structural alignments involving *Saccharomyces cerevisiae* Snf2 (PDB: 5X0Y), *S. cerevisiae* RSC (PDB: 6KW3 and 6TDA), and *S. cerevisiae* SWI/SNF (PDB: 6UXW) revealed high structural conservation of the ATPase/DNA translocase lobes—and beyond this, the domains that form the structural hub were highly conserved in their organization and position in bridging the two ATPase lobes. We note that a nucleotide analog (ADP-BeFx) is only present in the ySWI/SNF complex structure with the nucleosome (PDB: 6UXW), whereas all the other structures lack nucleotide. However, virtually no structural deviation is observed in the ATPase lobes or the structural hub when comparing the ySWI/SNF-nucleosome complex (containing ADP-BeFx) to the RSC structures lacking nucleotide.

Instead, a remarkable deviation was observed when comparing the hub in the Snf2-nucleosome complex (PDB: 5X0Y) to the hub in the SWI/SNF-nucleosome (PDB: 6UXW) or RSC-nucleosome complexes (PDB: 6KW3 and 6TDA). In the Snf2-nucleosome complex structure (lacking nucleotide), several hub domains were markedly shifted, which was most clearly visualized by comparing the structural alignment in the presence (Figure 6E1) or absence (Figure 6E2) of the Snf2-nucleosome structure. In the Snf2-nucleosome structure, ATPase lobe 1 remains fixed, whereas all hub domains (except for α2 and a portion of the post-HSA) and ATPase lobe 2 are shifted away from ATPase lobe 1 toward lobe 2, and lobe 2 itself shifts one base pair in the 3′ to 5′ direction along the DNA tracking strand, revealing a possible intermediate in the DNA translocation mechanism (Figure S6). This coincides with the opening of the cleft between the two ATPase lobes, within which ATP binding and hydrolysis occurs when the cleft is closed. The HSA domain in Snf2 and Sth1 is adjacent to the structural hub and is directly bound by the ARP module; notably, the ARPs themselves promote DNA translocation efficiency/coupling, and ARP deletions can be suppressed by certain hub domain mutations. In structures of full remodeler complexes, the cleft is closed. This raises the possibility that the ARPs (perhaps with other remodeler subunits) help constrain the position of the hub domains and promote a closed cleft conformation and/or act on the closed cleft to assist DNA translocation efficiency/coupling. This framework suggests that the alanine scanning, *mra*, and cancer-associated gain-of-function mutations residing in region 1, might likewise favor this closed cleft conformation—normally facilitated by the ARPs—to promote sliding or ejection.

Taken together, we utilized genetic, biochemical, and genomic studies, along with structural analysis, to define and functionally characterize a conserved structural hub in SWI/SNF-family remodelers that regulates coupling, and therefore DNA translocation efficiency by the two ATPase lobes, and interacts functionally with an ARP module. Surprisingly, all seven cancer-associated missense mutations that map to the regulatory region (region 1) of this hub displayed novel gain-of-function properties, which can now be understood in a structure-mechanism context.

Limitations of Study

In this work, we introduced cancer-associated mutations from the human BRG1 ATPase into its yeast ortholog, Sth1. Although these two ATPases are highly identical in the ATPase lobes and hub regions, individual amino acid substitutions may affect their function somewhat differently. However, as our conclusions are based on the similar behavior of several mutations in each domain, this concern is mitigated. Also, the behavior of certain hub mutations could, in principle, either be exacerbated or attenuated by other components in the full complex. Both of these possible caveats can be addressed through future work involving the reconstitution of recombinant SWI/SNF (BAF) family complexes. Our work shows genome-wide chromatin opening by cancer-associated gain-of-function mutations incorporated in yeast, and future work in human cells could determine whether these mutations cause either genome-wide or focal chromatin opening and if it occurs by enhancing sliding, or possibly ejection, in human cancers.

STAR★METHODS

Detailed methods are provided in the online version of this paper and include the following:

- KEY RESOURCES TABLE
- RESOURCE AVAILABILITY
  - Lead Contact
  - Materials Availability
  - Data and Code Availability
- EXPERIMENTAL MODEL AND SUBJECT DETAILS
- METHOD DETAILS
  - Recombinant Enzymes and Substrates
  - Biochemical Assays

- STH1 Genetics
- ATAC-Seq Method and Computational Analysis
- QUANTIFICATION AND STATISTICAL ANALYSIS

SUPPLEMENTAL INFORMATION

Supplemental Information can be found online at https://doi.org/10.1016/j.molcel.2020.09.024.

ACKNOWLEDGMENTS

We thank Tim Formosa, Alisha Schlichter, and Margaret Kasten for helpful comments on the manuscript. This work was supported by NIH/NCI R01 CA201396, NIH/NCI U54 232652, and Howard Hughes Medical Institute (HHMI), USA to B.R.C. and NIH CA24014 for core facilities.

AUTHOR CONTRIBUTIONS

C.R.C. generated/performed S/SAR reagents, chromatin substrates, biochemical assays, and structural alignment. N.V. performed genetic and ATAC-seq experiments. T.J.P. provided genomic data analysis. C.R.C., N.V., and B.R.C. contributed to the experimental design. C.R.C. and B.R.C. wrote the paper.

DECLARATION OF INTERESTS

The authors declare no competing interests.

Received: August 21, 2019
Revised: September 21, 2020
Accepted: September 22, 2020
Published: October 14, 2020

REFERENCES

Baker, R.W., Reimer, J.M., Carman, P.J., Arakawa, T., Dominguez, R., and Leschziner, A.E. (2020). Structural insights into assembly and function of the RSC chromatin remodeling complex. bioRxiv. https://doi.org/10.1101/2020.1103.1124.006361.

Bartholomew, B. (2014). Regulating the chromatin landscape: structural and mechanistic perspectives. Annu. Rev. Biochem. *83*, 671–696.

Becker, P.B., and Workman, J.L. (2013). Nucleosome remodeling and epigenetics. Cold Spring Harb. Perspect. Biol. *5*, a017905.

Buenrostro, J.D., Wu, B., Chang, H.Y., and Greenleaf, W.J. (2015). ATAC-seq: A Method for Assaying Chromatin Accessibility Genome-Wide. Curr. Protoc. Mol. Biol. *109*, 21–29.

Bultman, S.J., Gebuhr, T.C., and Magnuson, T. (2005). A Brg1 mutation that uncouples ATPase activity from chromatin remodeling reveals an essential role for SWI/SNF-related complexes in beta-globin expression and erythroid development. Genes Dev. *19*, 2849–2861.

Chen, X., Shen, Y., Draper, W., Buenrostro, J.D., Litzenburger, U., Cho, S.W., Satpathy, A.T., Carter, A.C., Ghosh, R.P., East-Seletsky, A., et al. (2016). ATAC-see reveals the accessible genome by transposase-mediated imaging and sequencing. Nat. Methods *13*, 1013–1020.

Clapier, C.R., and Cairns, B.R. (2012). Regulation of ISWI involves inhibitory modules antagonized by nucleosomal epitopes. Nature *492*, 280–284.

Clapier, C.R., and Cairns, B.R. (2014). Chromatin Remodeling Complexes. In Fundamentals of Chromatin, J.L. Workman and S.M. Abmayr, eds. (Springer), pp. 69–146.

Clapier, C.R., Längst, G., Corona, D.F., Becker, P.B., and Nightingale, K.P. (2001). Critical role for the histone H4 N terminus in nucleosome remodeling by ISWI. Mol. Cell. Biol. *21*, 875–883.

Clapier, C.R., Kasten, M.M., Parnell, T.J., Viswanathan, R., Szerlong, H., Sirinakis, G., Zhang, Y., and Cairns, B.R. (2016). Regulation of DNA Translocation Efficiency within the Chromatin Remodeler RSC/Sth1 Potentiates Nucleosome Sliding and Ejection. Mol. Cell *62*, 453–461.

Clapier, C.R., Iwasa, J., Cairns, B.R., and Peterson, C.L. (2017). Mechanisms of action and regulation of ATP-dependent chromatin-remodelling complexes. Nat. Rev. Mol. Cell Biol. *18*, 407–422.

Dutta, A., Sardiu, M., Gogol, M., Gilmore, J., Zhang, D., Florens, L., Abmayr, S.M., Washburn, M.P., and Workman, J.L. (2017). Composition and Function of Mutant Swi/Snf Complexes. Cell Rep. *18*, 2124–2134.

Dyer, P.N., Edayathumangalam, R.S., White, C.L., Bao, Y., Chakravarthy, S., Muthurajan, U.M., and Luger, K. (2004). Reconstitution of nucleosome core particles from recombinant histones and DNA. Methods Enzymol. *375*, 23–44.

Dykhuizen, E.C., Hargreaves, D.C., Miller, E.L., Cui, K., Korshunov, A., Kool, M., Pfister, S., Cho, Y.J., Zhao, K., and Crabtree, G.R. (2013). BAF complexes facilitate decatenation of DNA by topoisomerase IIα. Nature *497*, 624–627.

Flaus, A., Martin, D.M., Barton, G.J., and Owen-Hughes, T. (2006). Identification of multiple distinct Snf2 subfamilies with conserved structural motifs. Nucleic Acids Res. *34*, 2887–2905.

Funk, M., Niedenthal, R., Mumberg, D., Brinkmann, K., Rönicke, V., and Henkel, T. (2002). Vector systems for heterologous expression of proteins in Saccharomyces cerevisiae. Methods Enzymol. *350*, 248–257.

Han, Y., Reyes, A.A., Malik, S., and He, Y. (2020). Cryo-EM structure of SWI/SNF complex bound to a nucleosome. Nature *579*, 452–455.

He, S., Wu, Z., Tian, Y., Yu, Z., Yu, J., Wang, X., Li, J., Liu, B., and Xu, Y. (2020). Structure of nucleosome-bound human BAF complex. Science *367*, 875–881.

Helming, K.C., Wang, X., and Roberts, C.W.M. (2014). Vulnerabilities of mutant SWI/SNF complexes in cancer. Cancer Cell *26*, 309–317.

Ho, L., and Crabtree, G.R. (2010). Chromatin remodelling during development. Nature *463*, 474–484.

Hodges, H.C., Stanton, B.Z., Cermakova, K., Chang, C.Y., Miller, E.L., Kirkland, J.G., Ku, W.L., Veverka, V., Zhao, K., and Crabtree, G.R. (2018). Dominant-negative SMARCA4 mutants alter the accessibility landscape of tissue-unrestricted enhancers. Nat. Struct. Mol. Biol. *25*, 61–72.

Kadoch, C., Hargreaves, D.C., Hodges, C., Elias, L., Ho, L., Ranish, J., and Crabtree, G.R. (2013). Proteomic and bioinformatic analysis of mammalian SWI/SNF complexes identifies extensive roles in human malignancy. Nat. Genet. *45*, 592–601.

Kolde, R. (2019). Pretty Heatmaps. https://cran.r-project.org/web/packages/pheatmap/pheatmap.pdf.

Kovač, K., Sauer, A., Mačinković, I., Awe, S., Finkernagel, F., Hoffmeister, H., Fuchs, A., Müller, R., Rathke, C., Längst, G., and Brehm, A. (2018). Tumour-associated missense mutations in the dMi-2 ATPase alters nucleosome remodelling properties in a mutation-specific manner. Nat. Commun. *9*, 2112.

Liu, X., Li, M., Xia, X., Li, X., and Chen, Z. (2017). Mechanism of chromatin remodelling revealed by the Snf2-nucleosome structure. Nature *544*, 440–445.

Lorch, Y., and Kornberg, R.D. (2015). Chromatin-remodeling and the initiation of transcription. Q. Rev. Biophys. *48*, 465–470.

Lowary, P.T., and Widom, J. (1998). New DNA sequence rules for high affinity binding to histone octamer and sequence-directed nucleosome positioning. J. Mol. Biol. *276*, 19–42.

Narlikar, G.J., Sundaramoorthy, R., and Owen-Hughes, T. (2013). Mechanisms and functions of ATP-dependent chromatin-remodeling enzymes. Cell *154*, 490–503.

Parnell, T.J., Schlichter, A., Wilson, B.G., and Cairns, B.R. (2015). The chromatin remodelers RSC and ISW1 display functional and chromatin-based promoter antagonism. eLife *4*, e06073.

R Development Core Team (2018). R: A language and environment for statistical computing (R Foundation for Statistical Computing).

Saha, A., Wittmeyer, J., and Cairns, B.R. (2005). Chromatin remodeling through directional DNA translocation from an internal nucleosomal site. Nat. Struct. Mol. Biol. *12*, 747–755.

Schep, A.N., Buenrostro, J.D., Denny, S.K., Schwartz, K., Sherlock, G., and Greenleaf, W.J. (2015). Structured nucleosome fingerprints enable high-resolution mapping of chromatin architecture within regulatory regions. Genome Res. *25*, 1757–1770.

Schrödinger (2019). The PyMOL Molecular Graphics System, Version 2.3.2 (Schrödinger LLC).

Schubert, H.L., Wittmeyer, J., Kasten, M.M., Hinata, K., Rawling, D.C., Héroux, A., Cairns, B.R., and Hill, C.P. (2013). Structure of an actin-related subcomplex of the SWI/SNF chromatin remodeler. Proc. Natl. Acad. Sci. USA *110*, 3345–3350.

Schwanbeck, R., Xiao, H., and Wu, C. (2004). Spatial contacts and nucleosome step movements induced by the NURF chromatin remodeling complex. J. Biol. Chem. *279*, 39933–39941.

Shain, A.H., and Pollack, J.R. (2013). The spectrum of SWI/SNF mutations, ubiquitous in human cancers. PLoS ONE *8*, e55119.

Sikorski, R.S., and Hieter, P. (1989). A system of shuttle vectors and yeast host strains designed for efficient manipulation of DNA in Saccharomyces cerevisiae. Genetics *122*, 19–27.

Sokpor, G., Xie, Y., Rosenbusch, J., and Tuoc, T. (2017). Chromatin Remodeling BAF (SWI/SNF) Complexes in Neural Development and Disorders. Front. Mol. Neurosci. *10*, 243.

Szerlong, H., Hinata, K., Viswanathan, R., Erdjument-Bromage, H., Tempst, P., and Cairns, B.R. (2008). The HSA domain binds nuclear actin-related proteins to regulate chromatin-remodeling ATPases. Nat. Struct. Mol. Biol. *15*, 469–476.

Tate, J.G., Bamford, S., Jubb, H.C., Sondka, Z., Beare, D.M., Bindal, N., Boutselakis, H., Cole, C.G., Creatore, C., Dawson, E., et al. (2019). COSMIC: the Catalogue Of Somatic Mutations In Cancer. Nucleic Acids Res. *47* (D1), D941–D947.

Velankar, S.S., Soultanas, P., Dillingham, M.S., Subramanya, H.S., and Wigley, D.B. (1999). Crystal structures of complexes of PcrA DNA helicase with a DNA substrate indicate an inchworm mechanism. Cell *97*, 75–84.

Wagner, F.R., Dienemann, C., Wang, H., Stützer, A., Tegunov, D., Urlaub, H., and Cramer, P. (2020). Structure of SWI/SNF chromatin remodeller RSC bound to a nucleosome. Nature *579*, 448–451.

Wickham, H. (2016). ggplot2: Elegant Graphics for Data Analysis (Springer-Verlag).

Wilson, B.G., and Roberts, C.W. (2011). SWI/SNF nucleosome remodellers and cancer. Nat. Rev. Cancer *11*, 481–492.

Xia, X., Liu, X., Li, T., Fang, X., and Chen, Z. (2016). Structure of chromatin remodeler Swi2/Snf2 in the resting state. Nat. Struct. Mol. Biol. *23*, 722–729.

Yan, L., Wu, H., Li, X., Gao, N., and Chen, Z. (2019). Structures of the ISWI-nucleosome complex reveal a conserved mechanism of chromatin remodeling. Nat. Struct. Mol. Biol. *26*, 258–266.

Ye, Y., Wu, H., Chen, K., Clapier, C.R., Verma, N., Zhang, W., Deng, H., Cairns, B.R., Gao, N., and Chen, Z. (2019). Structure of the RSC complex bound to the nucleosome. Science *366*, 838–843.

Zhang, Y., Liu, T., Meyer, C.A., Eeckhoute, J., Johnson, D.S., Bernstein, B.E., Nusbaum, C., Myers, R.M., Brown, M., Li, W., and Liu, X.S. (2008). Model-based analysis of ChIP-Seq (MACS). Genome Biol. *9*, R137.

Zhou, C.Y., Johnson, S.L., Gamarra, N.I., and Narlikar, G.J. (2016). Mechanisms of ATP-Dependent Chromatin Remodeling Motors. Annu. Rev. Biophys. *45*, 153–181.

Zofall, M., Persinger, J., Kassabov, S.R., and Bartholomew, B. (2006). Chromatin remodeling by ISW2 and SWI/SNF requires DNA translocation inside the nucleosome. Nat. Struct. Mol. Biol. *13*, 339–346.

STAR★METHODS

KEY RESOURCES TABLE

REAGENT or RESOURCE	SOURCE	IDENTIFIER
Antibodies		
Anti-FLAG M2 (Mouse Monoclonal)	Sigma-Aldrich / Millipore Sigma	Cat#F3165; RRID: AB_259529
Anti-Mouse IgG HRP Conjugate (Goat Polyclonal)	Bio-Rad	Cat#170-6516; RRID: AB_11125547
Bacterial and Virus Strains		
E. coli BL21-CodonPlus(DE3)RIL	Agilent	Cat#230245
One Shot® MAX Efficiency® DH5α-T1R Competent Cells	Invitrogen	Cat#440097
One Shot TOP10 Chemically Competent *E. coli*	Invitrogen	Cat#C404010
Chemicals, Peptides, and Recombinant Proteins		
Nickel-NTA Agarose Beads	QIAGEN	Cat#30230
Anti-FLAG M2 Affinity Gel	Sigma-Aldrich	Cat#A2220
3x FLAG peptide	Sigma-Aldrich	Cat#F4799
HRV 3C protease	Novagen	Cat#71493-3
Slide-A-Lyser mini dialysis units	Thermo Scientific	Cat#69560
ATP, 100mM	GE Healthcare	Cat#27-2056-01
Glycogen, Ultra Pure	Thermo Scientific	Cat#10814010
Proteinase K, PCR grade	Roche Diagnostics	Cat#3115836001
Topoisomerase I, 500U	New England Biolabs	Cat#M0301L
*TDE1, Tagment DNA Enzyme,24RXN	Illumina	Cat#15027865
*Tagment DNA Buffer	Illumina	Cat#15027866
AMPure XP, 60 mL	Beckman Coulter	Cat#A63881
Minelute PCR purification kit	QIAGEN	Cat#28006
5-Fluoroorotic Acid (5FOA)	Toronto Research Chemicals	Cat#F59500
SsoAdvanced Universal SYBR Green Supermix	Bio-Rad	Cat#1725275
Lyticase from Arthrobacter luteus	Sigma-Aldrich / Millipore Sigma	Cat#L2524
Critical Commercial Assays		
*TDE1,Tagment DNA Enzyme,24RXN	Illumina	Cat#15027865
*Tagment DNA Buffer	Illumina	Cat#15027866
Deposited Data		
Raw Sequencing Data	This Paper	SRA: PRJNA561148
Raw Images	This Paper	Mendeley https://doi.org/10.17632/6x9cj9xjgk.1
Experimental Models: Organisms/Strains		
S. cerevisiae YBC3475	This Paper	N/A
S. cerevisiae BCY208	Gift from Dr. David Stillman	N/A
Oligonucleotides		
See Table S2 for oligonucleotides used in site-directed mutagenesis, and in sequencing	This paper (from IDT)	N/A
Recombinant DNA		
Plasmid BC3109: pCold TF-TetR-Sth1(301-1097)-Flag. WT.	Clapier et al., 2016	N/A

(*Continued on next page*)

Continued

REAGENT or RESOURCE	SOURCE	IDENTIFIER
Plasmid BC3108: pCDF. TetR-His7.	Clapier et al., 2016	N/A
Plasmid BC2565: pCDF. TetR-His7. TetR-(8 aa linker)-Sth1(301-1097)-Flag. WT.	Clapier et al., 2016	N/A
Plasmid BC1924: pRSF Duet.ARP9.ARP7	Clapier et al., 2016	N/A
Plasmid BC3356: pET Duet Rtt102-S-tag	Clapier et al., 2016	N/A
Plasmid BC3106: pUC12x200bp 601 NPS	Clapier and Cairns, 2012	N/A
Plasmid BC1312: pBluescript KS(-) & tetO site	Clapier and Cairns, 2012	N/A
Plasmid pBluescript	Clapier and Cairns, 2012	N/A
Plasmid p6/pRS314	Sikorski and Hieter, 1989	N/A
Plasmid p521/FB1521	Funk et al., 2002	N/A
Software and Algorithms		
ImageQuant TL	GE Healthcare	RRID: SCR_018374
ImageJ 1.52a (Java 1.8.0_112)	https://imagej.nih.gov/ij	RRID: SCR_003070
GraphPad Prism (Version 8.3.1)	GraphPad Software Inc	RRID: SCR_002798
PyMOL (Version 2.3.2)	Schrödinger, 2019; The PyMOL Molecular Graphics System, Schrödinger, LLC.	RRID: SCR_000305
Novoalign (Version 3.8.2)	Novocraft	RRID:SCR_014818
MultiRepMacsChIPSeq (Version 11.1)	https://github.com/tjparnell/biotoolbox	N/A
Macs2 (Version 2.1.1)	Zhang et al., 2008	N/A
BioToolBox package (Version 1.66)	TJ Parnell, Huntsman Cancer Institute (https://github.com/tjparnell/biotoolbox)	N/A
R (Version 3.5.2)	R Development Core Team, 2018	RRID: SCR_001905
GGPlot2	Wickham, 2016	RRID: SCR_014601

RESOURCE AVAILABILITY

Lead Contact

Further information and requests for resources and reagents should be directed to and will be fulfilled by the Lead Contact, Bradley R. Cairns (brad.cairns@hci.utah.edu).

Materials Availability

Plasmids generated in this study will be made available upon request.

Data and Code Availability

Original data for the biochemical assays and yeast genetics have been deposited to Mendeley Data: https://doi.org/10.17632/6x9cj9xjgk.1. The accession number for the raw genomics data reported in this paper is SRA: PRJNA561148.

EXPERIMENTAL MODEL AND SUBJECT DETAILS

The S. cerevisiae strain YBC3475 (*sth1Δ*) was created in our lab, and the strain BCY208 (*WT*) was a gift from Dr. David Stillman. *S. cerevisiae* strains were stored as glycerol stocks at -80°C. Before use, the strains were revived on YPD plates at 30°C for 48-72 hours. All yeast cultures and plates were grown at 30°C.

METHOD DETAILS

Recombinant Enzymes and Substrates

Remodelers

The complexes containing Sth1 protein alone (S) were expressed in *Escherichia coli* BL21-CodonPlus(DE3)RIL, upon 0.5 mM isopropyl-β-D-thiogalactoside induction at OD_{600nm} ~0.5 for 24 h at 15°C, as complexes from two vectors: one –pCDFDuet-1- bears a gene coding for TetR-$(His)_7$, and the other –pCold TF (Takara) lacking the His tag, is a gene fusion containing TF-3C cleavage site-TetR-$Sth1_{(301-1097)}$-Flag. A sequence of eight amino acids (GGQGGQGG) was inserted between the genes coding for TetR and Sth1. The

trigger factor (TF) fusion is necessary for the solubility of Sth1 alone; and the Tet repressor DNA-binding domain (TetR) enables *tetO* binding for the DNA translocation assay.

The complexes containing Sth1 protein, with the Arp7, Arp9, and Rtt102 proteins (SAR) were produced by co-expression in *E. coli* BL21-CodonPlus(DE3)RIL (auto-induction in ZYC media), from three Duet vectors: the first, pCDFDuet-1, contains genes coding for TetR-Sth1$_{(301-1097)}$-Flag (with a sequence of eight amino acids GGQGGQGG inserted between TetR and Sth1) and TetR-(His)$_7$; the second, pRSFDuet-1, bears *ARP7* and *ARP9* genes; the third, pETDuet-1, contains the gene coding for Rtt102-S-tag.

All S/SAR properly assembled complexes containing a heterodimer of TetR were obtained by two successive affinity purifications. The bacteria-cell extracts were first mixed with nickel-nitrilotriacetic acid agarose resin (QIAGEN), capturing the complex (and the unwanted TetR homodimer). The sample eluted from the resin was further purified using the Flag tag and anti-Flag M2 affinity gel and eluted with 3x Flag peptide (Sigma) to obtain the desired complex. For Sth1 alone (S), trigger factor (TF) was cleaved from the complex using human rhinovirus 3C protease (Novagen) using 1 U of protease per 0.1 mg of complex by incubating 16 h at 4°C, in the presence of 10 mM β-mercaptoethanol and 0.5 mM EDTA. Finally, the purification of S/SAR complexes was achieved by gel-filtration on two S200GL 10/300 (Amersham, GE) in series. Obtained complexes were homogeneous and monodisperse.

Nucleosomes

Histone octamers were produced from single recombinant *Drosophila* histones expressed in *E. coli* BL21-CodonPlus(DE3)RIL, purified as inclusion bodies, and assembled in octamers by salt-dialysis, essentially as described in Dyer et al. (2004).

The 200-bp DNA fragments containing centrally-located 601 strong positioning sequence (Lowary and Widom, 1998) were produced (as described in Clapier and Cairns, 2012) from plasmids pUC12x601 digested by AvaI and purified from the backbone using preparative electrophoresis (PrepCell, Bio-Rad) 4.5% (37.5:1) native polyacrylamide gel running at 400V constant in 0.5x TBE (Tris-Borate 45 mM, pH 8, with 1 mM EDTA) with TE (Tris 10 mM, 1 mM EDTA) as elution buffer.

Mononucleosome assemblies were performed (as described in Clapier and Cairns, 2012) by titration reactions, as 50 μl reactions containing 40 pmol of DNA fragment (0.8 μM) in 2 M KCl mixed with a variable amount of histone octamers covering the equimolar ratio, with a linear salt-gradient dialysis applied from 2 M to 50 mM KCl in cold conditions (essentially as described in Dyer et al., 2004), using an Econo-Pump (Bio-Rad) and Slide-A-Lyser Mini Dialysis units with a 7,000 molecular weight cutoff (Thermo Scientific).

Nucleosome arrays were assembled as 100 μl reactions containing 5 μg of pBluescript plasmid DNA mixed with 23 pmol of histone octamers in 2 M KCl, NEB4 buffer 1x, 0.1 mg.ml^{-1} BSA, in the presence of 10 U of Topoisomerase I (NEB) with a linear salt-gradient dialysis applied from 2 M to 50 mM KCl at 30°C, using an Econo-Pump (Bio-Rad) and Slide-A-Lyser Mini Dialysis units with a 7,000 molecular weight cutoff (Thermo Scientific).

Biochemical Assays

ATPase Assay

Measurement of ATP hydrolysis by a colorimetric assay was based on the formation of a complex between inorganic phosphate and molybdate-malachite green. ATPase assays were performed (as described in Clapier and Cairns, 2012) at 30°C after 30 min incubation of 10 pmol of S/SAR complexes (0.4 μM) with 500 ng of pBluescript plasmid in 10 mM HEPES buffer [pH 7.3], 20 mM potassium acetate, 5 mM $MgCl_2$, 0.5 mM dithiothreitol, 0.1 mg.ml^{-1} BSA and 5% glycerol, in the presence of 1 mM ATP. After 30 min incubation at 30°C with 500 rpm shaking in a Thermomixer (Eppendorf), 800 μl of MGAM reagent (3 volumes of MG = 0.045% (w/v) malachite green in 0.1 N HCl, mixed with 1 volume of AM = 4.2% (w/v) ammonium molybdate green in 4 N HCl) were added, followed 1 min later by 100 μl of 34% (w/v) Na_3citrate. Measurements were performed at OD_{650nm} 10 min later.

DNA-Translocation Assay

The DNA-translocation assay measured plasmid supercoils generated by a single S/SAR protein anchored through its TetR fusion to a previously relaxed (by *E. coli* topoisomerase I) plasmid DNA containing a single *tetO* operator sequence. Translocation by tethered Sth1 along the DNA backbone creates positive supercoils in front of the translocase, and negative supercoils behind, but *E. coli* Topoisomerase I relaxes only negative supercoils. Thus, translocation yields one positive supercoil / 10 bp translocation. Time-course experiments were performed (essentially as described in Clapier et al., 2016) using a 100 μl starting reaction contained 5 pmol of S/SAR proteins (50 nM) in the presence of 2.5 μg of previously relaxed plasmid, 1 mM ATP, 12.5 U of topoisomerase I (NEB) in NEB4 1x buffer, 1mg.ml^{-1} BSA. All reactions were incubated at 30°C, and 20 μl aliquots were removed at each time point for a 20 min heat inactivation at 65°C. Deproteinization was performed by adding 2 μl of proteinase K at 10 mg.ml^{-1} and 1 μl of SDS 20% and incubated at 50°C for 1h. Samples were subsequently precipitated in ethanol, prior to loading on a 1.3% agarose gel run for 3h at 130V constant. Gels were stained for 20 min in a 1 μg.ml^{-1} ethidium bromide solution and scanned on a Typhoon Trio (Amersham, GE).

Nucleosome Sliding Assay

Time-course sliding assays were performed (essentially as described in Clapier and Cairns, 2012) using 1:2 enzyme: substrate ratio with a 50 μl starting reaction containing 500 fmol of S/SAR protein complexes (10 nM) in the presence of 1 pmol of mononucleosomes (20nM) and incubated in 10 mM Tris buffer [pH 7.4], 50 mM KCl, 3 mM $MgCl_2$, 0.1 mg.ml^{-1} BSA, 1 mM ATP at 30°C with shaking at 500 rpm in a Thermomixer (Eppendorf). 10 μl aliquots were removed at each time point and reactions were stopped by adding 200 ng competitor DNA (excess of pBluescript plasmid) and incubated for an additional 30 min at 30°C with shaking at 500 rpm in a Thermomixer. Samples were loaded using 10% glycerol on a 4.5% (37.5:1) native polyacrylamide gel and run in 0.4x TBE for 55 min at 110 V constant. Gels were stained for 10 min in a 1 μg.ml^{-1} ethidium bromide solution and scanned on a Typhoon Trio (Amersham, GE).

Nucleosome Ejection Assay

Nucleosome ejection assays were performed (essentially as described in Clapier et al., 2016) in a 50 μl reaction by incubating 1 pmol of S/SAR protein complexes (20 nM) in the presence of 500 ng of nucleosome arrays in 10 mM Tris buffer [pH 7.4], 50 mM KCl, 3 mM $MgCl_2$, 0.1 mg.ml^{-1} BSA, 1 mM ATP at 30°C with shaking at 500 rpm in a Thermomixer (Eppendorf) for 90 min. Deproteinization was performed by adding 5 μl of proteinase K at 10 mg.ml^{-1} and 2.5 μl of SDS 20% and incubated at 50°C for 1h. Samples were subsequently precipitated in ethanol, prior to two-dimensional separation on a 1.3% agarose gel as described in Clapier et al. (2001). Gels were stained for 15 min in a 1 μg.ml^{-1} ethidium bromide solution and scanned on a Typhoon Trio (Amersham, GE).

STH1 Genetics

Complementation Assay

Complementation was assessed by the ability of plasmid-bourne *sth1* mutant derivatives to complement an *sth1Δ* mutation, in a plasmid shuffle format. To generate the construct used, a KpnI/EcoRI-digested yeast genomic fragment containing *STH1* under its own genomic promoter was cloned into a p6/pRS314 backbone plasmid (Sikorski and Hieter, 1989). To test whether a *sth1* mutant complements the loss of WT *STH1*, the plasmid pRS314 (empty backbone control), *WT* positive control and mutant *sth1* (generated by standard site directed mutagenesis of the WT plasmid) were transformed into the plasmid-covered *sth1Δ S. cerevisiae* S288C strain, YBC3475 ($lys^{2\text{-}128\delta}$ $leu^{2\Delta 1}$ $ura^{3\text{-}52}$ $trp1^{\Delta 63}$ $his3^{\Delta 200}$ *sth1Δ::HIS3* [pRS316 $STH1^{WT}$]). Ten-fold serial dilutions of the transformants were spotted on media lacking tryptophan (SC-TRP), which selects for the above new plasmids, and on the SC-TRP plates with added 5-fluoroorotic acid (SC-TRP+5FOA) to select for the loss of the WT copy of *STH1* (pRS316 $STH1^{WT}$). The spotted plates were incubated at 30°C and imaged.

Dominant Lethality Assay

Dominant lethality was assessed by expression of Sth1 derivatives, using the methionine-regulated *MET25* promoter, in the presence of WT *STH1*. N-terminally 2xFLAG and C-terminally 7xHis tagged STH1 gene was cloned into the backbone plasmid, p521/FB1521 (Funk et al., 2002) to generate *MET25* promoter-driven *WT STH1* plasmid. Mutant *sth1* plasmids were created via standard site directed mutagenesis via PCR.

To test for dominant lethality, the plasmid p521 (empty backbone control), *WT* and mutant *sth1* were transformed into the *Saccharomyces cerevisiae* W303 strain, BCY208 ($ade^{2\text{-}1}$ $can^{1\text{-}100}$ $his^{3\text{-}11}$ $leu^{2\text{-}1}$ $trp^{1\text{-}1}$ $ura^{3\text{-}1}$). The transformed strains were grown and ten-fold serial dilutions of each were spotted on synthetic complete (SC)-agar media plates lacking uracil (SC-URA) to select for the transformed plasmid and SC-agar plates lacking uracil, methionine and cysteine (SC-URA-MET-CYS) to induce the MET25 promoter dependent expression from the transformed plasmid. The plates were grown at 30°C for 48 hours. The assay was performed in triplicate.

ATAC-Seq Method and Computational Analysis

ATAC-seq Method

A modified ATAC-seq protocol was established using multiple published methods to assess chromatin accessibility in formaldehyde-fixed *Saccharomyces cerevisiae* nuclei (Buenrostro et al., 2015; Chen et al., 2016; Schep et al., 2015). In brief, *WT* and mutant *STH1* plasmids used for the dominant lethality assay were transformed in BCY208. Multiple colonies of transformants were used as inoculum for each strain grown at 30°C in synthetic defined (SD) media without inducing overexpression. After 6 hours, cells were pelleted and re-suspended in inducing SD media (lacking methionine and cysteine) and grown for 1.5 hours at 30°C. Cells were fixed in 1% formaldehyde for 15 min and quenched with 0.125 M glycine for 20 min at room temperature. A total of 5 million cells per library were harvested (Schep et al., 2015), washed thrice in PBS and stored at −80°C before further processing. The cells were thawed on ice, spheroplasted, resuspended in lysis buffer (10 mM Tris-HCl [pH 7.4], 10 mM NaCl, 3 mM $MgCl_2$, 0.05% IGEPAL CA-630), and pelleted immediately. The crude nuclear pellets generated were put through a 30-minute transposition reaction using 2 μl TDE1, Tagment DNA Enzyme (Illumina) as described in earlier work (Buenrostro et al., 2015). Crosslink reversal was carried out overnight followed by DNA purification as described previously (Chen et al., 2016). Purified DNA was used to prepare libraries, which were put through a clean-up and size-selection step using AMPure XP beads, and then sequenced with 50bp paired-end reads on an Illumina NovaSeq 6000.

Computational Analysis

Sequences were aligned to the *S. cerevisiae* genome (SGD release 64, UCSC SacCer3) using Novocraft Novoalign (version 3.8.2), allowing for one random alignment for multi-mapping reads and specifying sequence adapters for trimming during alignment. Alignments were analyzed using the MultiRepMacsChIPSeq pipeline (version 11.1) (https://github.com/HuntsmanCancerInstitute/MultiRepMacsChIPSeq). Alignments overlapping a blacklist of high-copy regions (primarily telomeric and rDNA loci, plus mitochondrial DNA) were excluded from further analysis. Optical duplicates were removed (pixel distance of 12000) and coordinate duplicates were subsampled to a uniform 8% level across samples (starting duplication rates ranged from 5% to 17%, mean 13%). For cut-site analysis, fragments were treated as single-end, and a coverage track generated by shifting 5′ coordinates by −10 bp and extending 20 bp (effectively 10 bp flanking the cut site). Sample replicate coverage tracks were depth-normalized by scaling to Reads Per Million and averaged. A ratio of signal over the chromosomal mean signal was generated using Macs2 (version 2.1.1) (Zhang et al., 2008).

The mean signal across the protein-coding gene Transcription Start Site (TSS), from −500 to +500 bp, were collected using *get_datasets* from the BioToolBox package (version 1.66) (https://github.com/tjparnell/biotoolbox). Profile data over the TSS was collected using the BioToolBox application *get_relative_data*, over −500 to +500 bp in 20 bp windows, excluding windows overlapping neighboring genes. For plotting, genes were ordered by decreasing promoter NDR length, as determined by nucleosome mapping (Parnell et al., 2015). Only one gene in a divergent promoter pair is included in the plot. PCA plots, average TSS profile plots, and heatmaps were generated with standard R (version 3.5.2) (R Development Core Team, 2018) scripts, *GGPlot* (Wickham, 2016) and *pHeatmap* (Kolde, 2019) packages.

QUANTIFICATION AND STATISTICAL ANALYSIS

For DNA translocation assays, nucleosome sliding assays and nucleosome ejection assays, representative gels from multiple experiments are shown in Figures 2, 3, 4, and S2. Quantification of the ATPase activity was performed in at least duplicates to ensure consistency. Coupling value calculations were performed at least twice, and the values presented in the figures are representative and correspond to the gel images depicted above them.

All *Saccharomyces cerevisiae* genetic assays (complementation and dominant lethality assays) were performed in triplicates, imaged, and compared. One representative image, from the set of three, for each mutant in the two assays, is provided in Figures 2, 3, 4, and S2.

The cut-offs and statistical analysis required for the ATAC-seq computational analysis are described in Method Details.

Molecular Cell, Volume *80*

Supplemental Information

Cancer-Associated Gain-of-Function Mutations

Activate a SWI/SNF-Family Regulatory Hub

Cedric R. Clapier, Naveen Verma, Timothy J. Parnell, and Bradley R. Cairns

SUPPLEMENTAL INFORMATION

Cancer-Associated Gain-of-Function Mutations Activate a SWI/SNF-Family Regulatory Hub

Cedric R. Clapier,[1,2,3,*] Naveen Verma,[1,2,3] Timothy J. Parnell,[2] and Bradley R. Cairns[1,2,*]

[1]Department of Oncological Sciences and Howard Hughes Medical Institute, University of Utah School of Medicine, Salt Lake City, UT 84112, USA
[2]Huntsman Cancer Institute, University of Utah School of Medicine, Salt Lake City, UT 84112, USA
[3]These authors contributed equally
[*]Correspondence: brad.cairns@hci.utah.edu or cedric.clapier@hci.utah.edu
Lead Contact: brad.cairns@hci.utah.edu

Figure S1, related to Figures 2, 3 and 4.
Schematic of DNA Translocation Assays.

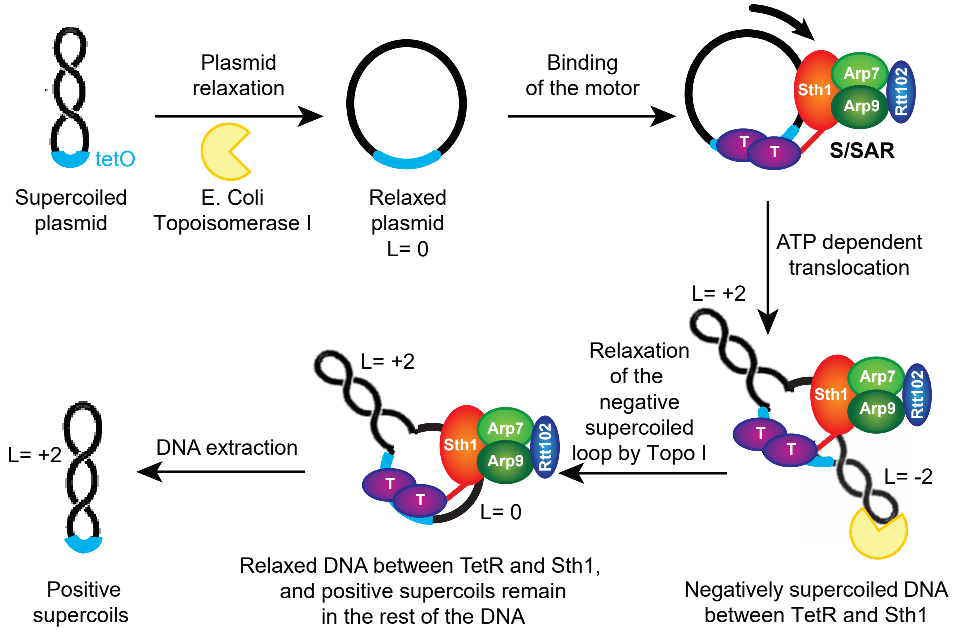

Principle of the tethered translocation assay. Supercoiled topoisomers are proportional to DNA translocation by a tethered Sth1. Sth1 is fused to the Tet repressor (T-Sth1), which forms a heterodimer with an unfused Tet repressor (T). A Tet-Sth1 complex, with (**SAR**) or without (**S**) the Arp7-Arp9-Rtt102 module, anchors/tethers to a single *tetO* operator present on a plasmid DNA, which has previously been relaxed by *E. coli* Topoisomerase I. The ATPase domain of Sth1 engages the DNA and performs ATP-dependent DNA translocation. Translocation of Sth1 along the DNA backbone generates negative supercoils between the enzyme and the *tetO* anchor, and positive supercoils in front of the enzyme. The negative supercoils are specifically relaxed by *E. coli* Topoisomerase I, resulting in the accumulation of positive supercoils that can be visualized after deproteinization on an agarose gel.

Figure S2, related to Figures 2 and 6.
Alanine Scanning, and Addition of the ARP Module.

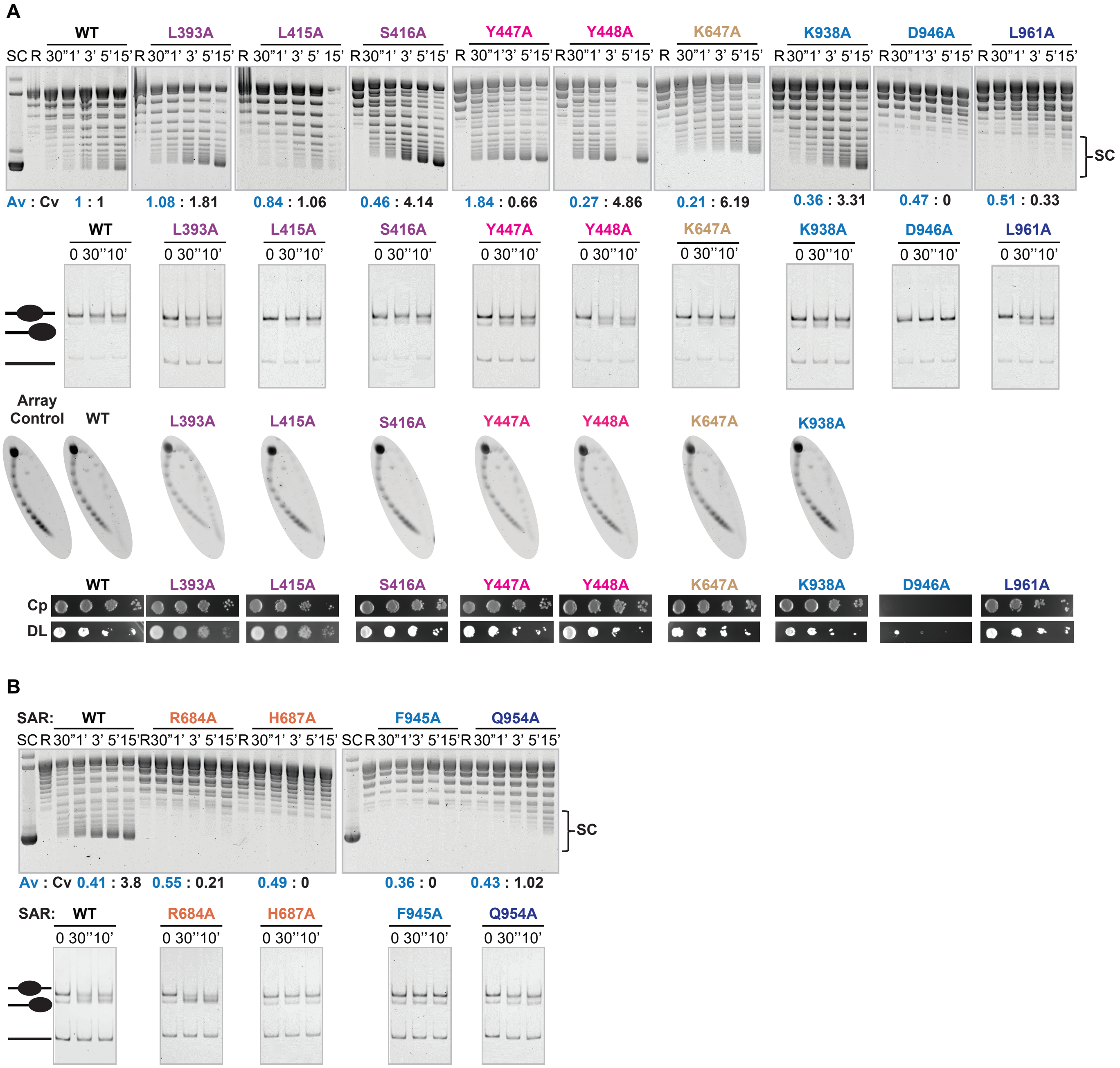

(A) Comparative impact of additional alanine substitutions either enhancing or abolishing DNA translocation, coupling, and nucleosome sliding, confirming two distinct functional regions within the hub. All assays performed and results depicted as in Figure 2A.
(B) First row: Comparative DNA translocation and ATPase activity by Sth1 derivatives in the SAR format. Addition of the ARP module to Sth1 (SAR) enhances DNA translocation for mutations R684A and Q954A, but not H687A and F945A. The plasmid stimulated ATPase activity/value (Av, blue text) and coupling value (Cv, black text; expressed as supercoiling/ATPase hydrolyzed) and normalized to WT Sth1 alone (Av:Cv=1:1). SC: highly-supercoiled topoisomers, R: relaxed plasmid.
Second row: Comparative sliding of mononucleosomes performed using SAR derivatives. Addition of the ARP module to Sth1 (SAR) enhances nucleosome sliding for mutations R684A and Q954A, but not H687A and F945A.

Figure S3, related to Figures 3 and 4.
A scatter plot comparing the biochemical properties (ATPase values and Coupling values) of Sth1 mutations groups/clusters the mutants into three categories: loss-of-function, gain-of-function and dominant lethal.

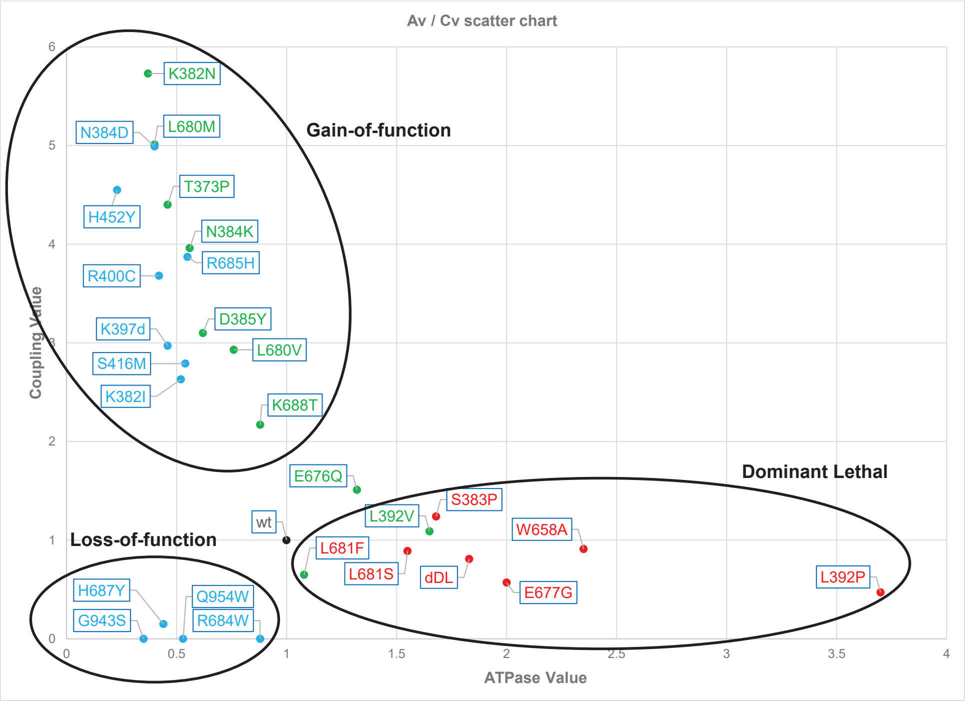

Scatter plot of the ATPase / Coupling values from various mutations. Cancer-associated mutations are depicted with blue markers and labels. Mutations compensating for the lack of the ARPs (*mra*), and not dominant lethal, are depicted with green markers and labels. Mutation L681F, also compensating for the lack of the ARPs but dominant lethal, is depicted with a green marker, but red label. Mutations that are dominant-lethal and are unable to complement *sth1*Δ, including W658A, are depicted with a red marker and label. Notably, cancer-associated mutations partitioned into two distinct clusters corresponding to the loss-of-function and the gain-of-function mutations: a first cluster characterized by a lowering of the ATPase activity and a loss of coupling when compared to wild-type (wt; ATPase and coupling values both normalized to 1.0 for wild-type) grouped the loss-of-function cancer-associated mutations. In contrast, a second cluster, while still displaying reduced ATPase activity, presented an increase in coupling and grouped the gain-of-function cancer-associated mutations. Of note, the vast majority of the *mra* mutations belonged to this cluster. Finally, a third cluster characterized by an increase of the ATPase activity without change in coupling, contained the mutations that are dominant-lethal. Remarkably, the three clusters defined here by the scatter plot of the ATPase and coupling values matched the three clusters generated by the hierarchical clustering of the ATAC-seq samples (Figure 5).

Figure S4, related to Figure 5.
ATAC-seq Accessibility, Mutant Sth1 Incorporation, and Heat Map.

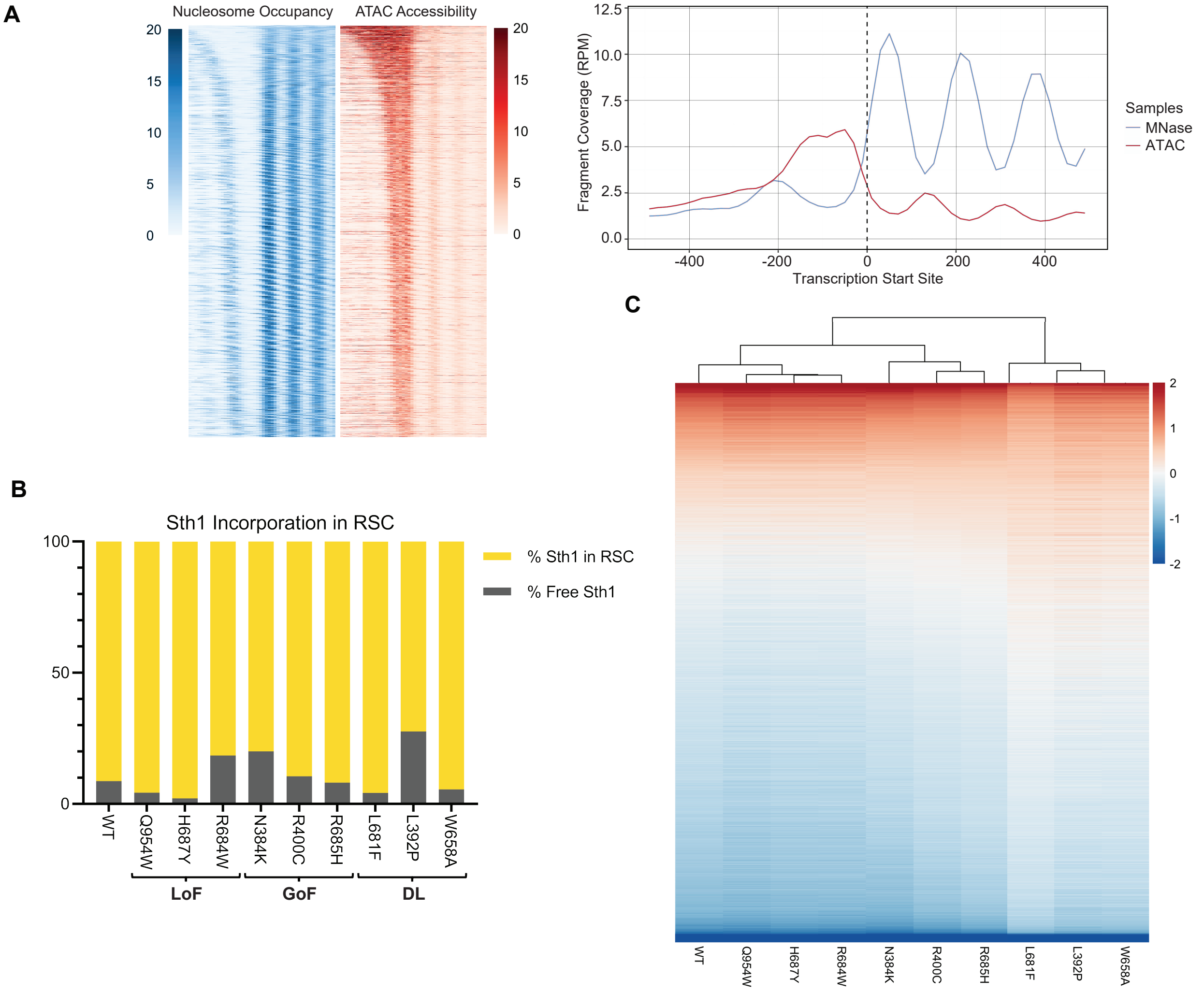

(A) **Nucleosome occupancy comparisons to ATAC accessibility.** Heat maps of signal from wild type strains around the TSS for protein-coding genes (rows), ordered by the decreasing length of the promoter NDR. The nucleosome occupancy represents the signal from MNase fragment coverage (recording the central 75 bp of each fragment). The signal indicates strong (dark blue) or weak (light blue) nucleosome occupancy. The ATAC accessibility represents the fragment coverage (20 bp) encompassing each observed ATAC cut site. The signal indicates the strong (dark red) or weak (light red) accessibility of DNA not wrapped in nucleosome and/or proteins. On the right is a graph of the mean profile signal density for both nucleosome occupancy and ATAC-seq.

(B) **Mutant Sth1 incorporation in RSC complex.** *S. cerevisiae* cells were transformed and grown as described in methods for ATAC-seq, cells were lysed, and the whole cell extract immunoprecipitaed with α-Flag resin. The bound fraction was eluted, run on a non-denaturing protein gel (Invitrogen NativePAGE Bis-Tris Gel System), and probed with α-FLAG to detect the slow migrating RSC (with mutant Sth1 incorporated) and the faster migrating free mutant Sth1 subunit present in the whole cell lysate. The bands intensities corresponding to slow migrating RSC and faster-migrating free Sth1 were quantified using ImageJ. The percentage of free Sth1 (in gray) and Sth1 incorporated into RSC (in yellow) for WT and each mutant were plotted as a bar graph.

(C) **Mean accessibility heat map over 1 kb of all TSS, and derived hierarchical clustering tree.** Log2 fold ratio of signal over background was collected in a 1 kb window (-500 to +500 bp) of protein-coding gene TSS and plotted as a heat map.

Figure S5, related to Figure 5.
Gallery of illustrative snapshots from a genome browser.

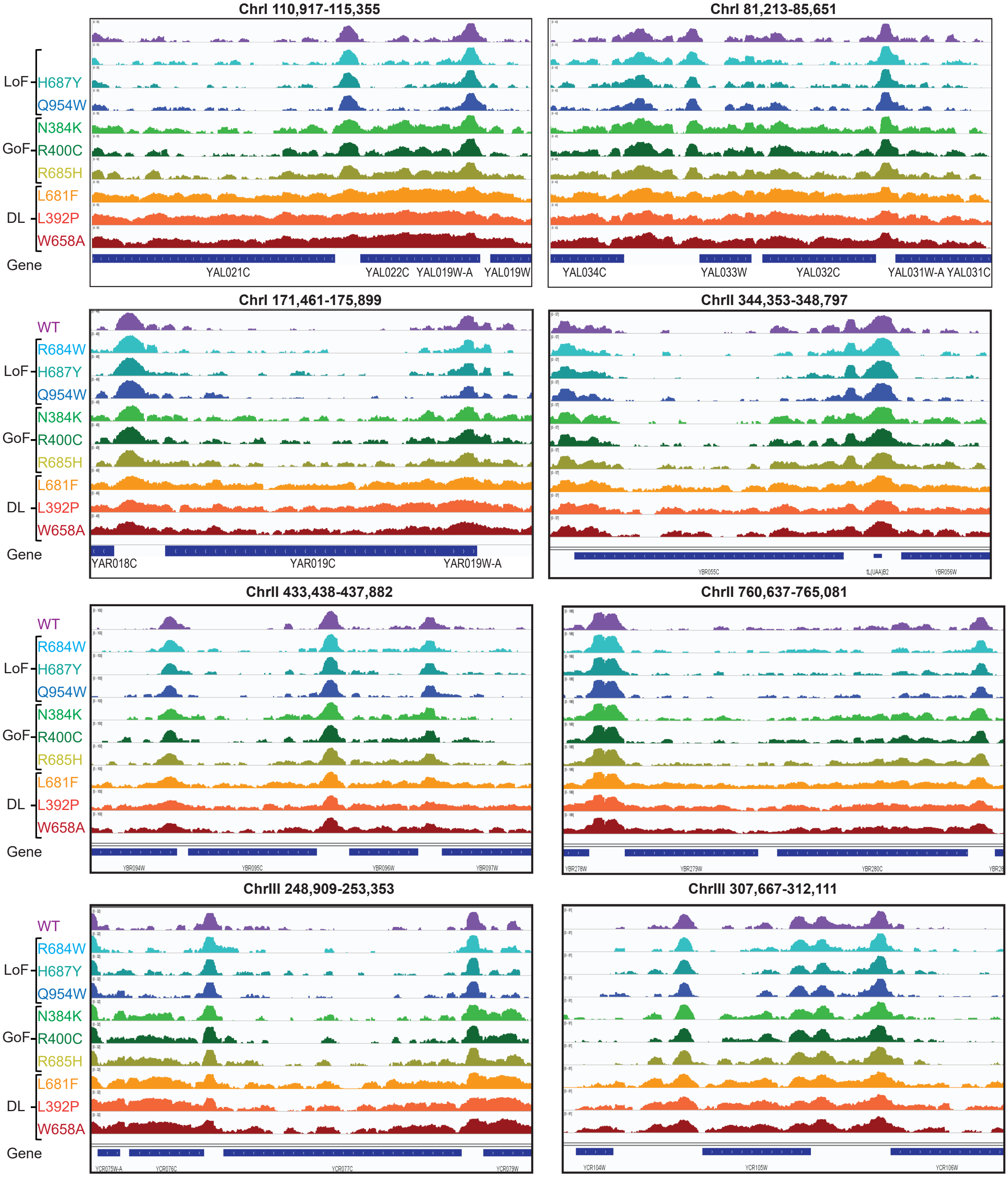

Snapshots from a genome browser showing the ATAC-seq accessibility coverage (Log RPM) across the chromosome. All tracks within each snapshot are scaled to the same Y axis. Progressive/increasing impact is generally observed as one examines the loss-of-function, gain-of-function cancer-associated missense, and dominant lethal mutations.

Figure S6, related to Figure 6.

In the context of the Snf2 ATPase alone (lacking the ARP module and other subunits), the position of most of the hub domains are shifted, along with a 3' to 5' one base-pair shift in the positioning of the ATPase lobe 2 with respect to lobe 1, opening the ATP binding/hydrolysis cleft, revealing a possible intermediate conformation in the DNA translocation mechanism.

A

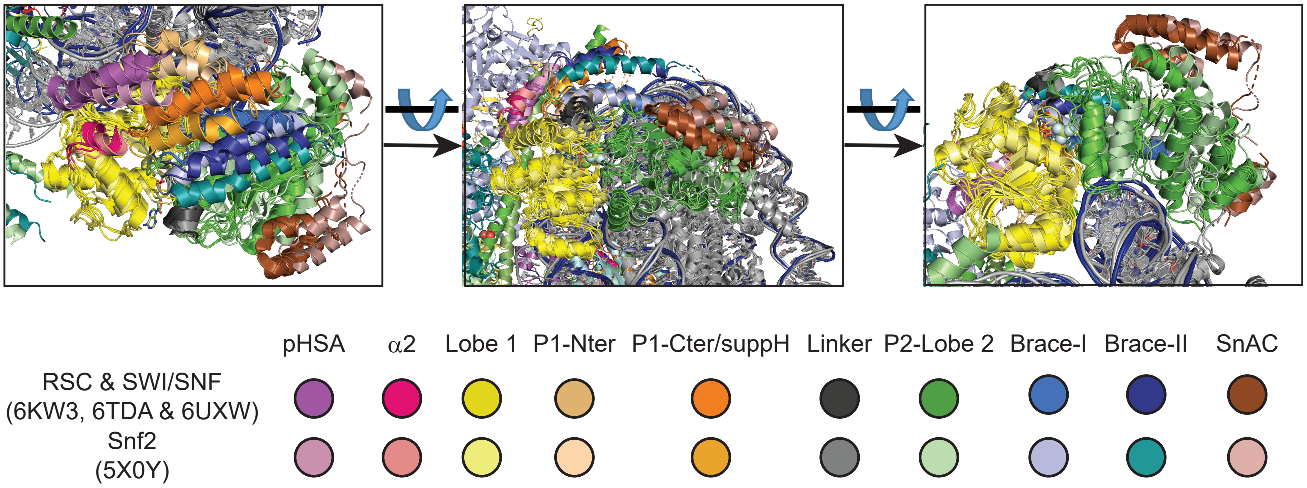

B

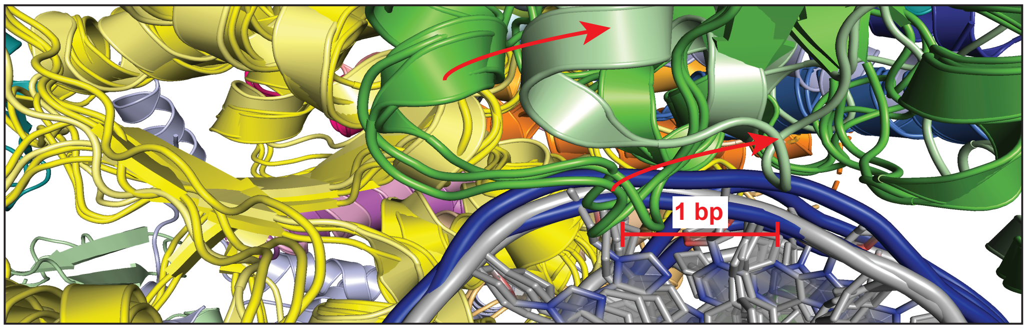

(A) Structural alignment of various Remodeler-nucleosome complexes (PDB ID 5X0Y, 6KW3, 6TDA and 6UXW) based on the alignment of their respective histone octamers reveals that, in the absence of the ARP module and other subunits, there is a structural shift of many hub domains, along with a structural shift of the ATPase lobe 2 away from lobe 1. The different views represent snapshots on a sequential rotation of the initial alignment on the left. Domains of the RSC & SWI/SNF Remodeler-nucleosome complexes (PDB ID 6KW3, 6TDA, 6UXW) and the Snf2-nucleosome complex (PDB ID5X0Y) are color-coded according to the depicted entry key below. Structural alignment has been obtained using The PyMOL Molecular Graphics System, Version 2.3.2 Schrödinger, LLC.

(B) Magnification of the right view of panel (A) showing that the shift in positioning of lobe 2 with respect to lobe 1 (red arrows) matches the distance of one base-pair along the bound DNA (blind-ended red line), revealing a possible intermediate conformation in the DNA translocation mechanism. Domains are color-coded as in (A).

Table S1, related to Figures 2, 3, 4, 5, and 6.

All mutations investigated with biochemistry, genetic and genomics data presented together along with functional classification.

Hub Domain	Sth1 Residue & Mutation	DNA Translocation	ATPase value	Coupling value	Mono-nuc. Sliding	Array-nuc. Ejection	Genet. Cp	Genet. DL	ATAC -seq	GoF, LoF. & DL	Hub Region
HSA-Cter	T373P	✓↑	↓	↑↑	?	?	✓	×		GoF	#1
	K382N	✓↑	↓	↑↑	?	?	✓	×		GoF	#1
	K382I	✓↑	↓	↑↑	✓↑	✓	✓	×		GoF	#1
	S383P	✓↑	↑	↑	?	?	×	✓		GoF+DL	#1
	N384K	✓↑	↓	↑↑	?	?	✓	×	✓↑	GoF	#1
	N384D	✓↑	↓	↑↑	✓↑	✓	✓	×		GoF	#1
	D385Y	✓↑	↓	↑↑	?	?	✓	✓		GoF	#1
	L392V	✓↑	↑	=	✓ (*)	✓↑ (*)	✓	× (*)		GoF	#1
	L392P	✓↑	↑↑	↓↓	✓ (*)	✓↑↑ (*)	×	✓ (*)	✓↑↑	GoF+DL	#1
	ΔDL	✓↑	↑	↓	✓ (*)	✓↑ (*)	×	✓ (*)		GoF+DL	#1
pHSA	L393A	✓↑	=	↑	✓↑	✓↑	✓	×		GoF	#1
	D394A	✓↑	↓	↑↑	✓↑	✓	✓	×		GoF	#1
	K397A	✓↑	↓	↑↑	✓↑	×	✓	×		GoF	#1
	K397Δ	✓↑	↓	↑↑	✓↑	✓	×	×		GoF	#1
	R400A	✓↑	↓	↑↑	✓↑	✓	✓	×		GoF	#1
	R400C	✓↑	↓	↑↑	✓↑	✓	✓	×	✓↑	GoF	#1
	L415A	✓=	↓	=	✓=	✓	✓	×		GoF	#1
	S416A	✓↑	↓	↑↑	✓=	×	✓	×		GoF	#1
	S416M	✓↑	↓	↑↑	✓↑	✓	✓	×		GoF	#1
α2	Y447A	✓↑	↑	↓	✓↑	✓↑	✓	×		GoF	#1
	Y448A	✓↑	↓↓	↑↑	✓↑	✓	✓	×		GoF	#1
	H452A	✓↑	↓↓	↑↑	✓=	×	✓	×		GoF	#1
	H452Y	✓=	↓↓	↑↑	✓↑	✓	✓	×		GoF	#1
P1-Nter	K647A	✓=	↓↓	↑↑	✓=	✓	✓	×		GoF	#1
	F655A	✓=	↓	↑	✓↑↑	✓	✓	×		GoF	#1
	W658A	✓↑	↑	=	✓↑↑	✓↑↑	×	✓	✓↑↑	GoF+DL	#1
	F659A	✓↑	↓	↑	✓↑↑	✓↑	✓	✓		GoF	#1
P1-Cter / suppH	E676Q	✓↑	↑	↑	✓ (*)	✓↑ (*)	✓	× (3)		GoF	#1
	E677G	✓=	↑↑	↓	?	?	×	✓		GoF+DL	#1
	L680M	✓↑	↓	↑↑	?	?	✓	×		GoF	#1
	L680V	✓↑	↓	↑↑	?	?	✓	×		GoF	#1
	L681F	✓=	=	↓	✓ (*)	✓ (*)	✓	✓ (3)	✓↑↑	GoF+DL	#1
	L681S	✓↑	↑	↓	?	?	✓	✓		GoF+DL	#1
	R684A	× (1)	↓	~0	× (1)	×	✓	×		LoF	#2
	R684W	×	↓	0	×	×	×	×	✓=	LoF	#2
	R685A	✓↑	↓	↑	✓↑	✓↑	✓	×		GoF	#1
	R685H	✓↑	↓	↑↑	✓↑	✓↑	✓	×	✓↑	GoF	#1
	H687A	× (2)	↓	0	× (2)	×	×	✓		LoF	#2
	H687Y	×	↓↓	~0	×	×	×	✓	✓=	LoF	#2
	K688A	✓↑	↓	↑↑	✓=	×	✓	×		GoF	#1
	K688T	✓↑	↓	↑↑	?	?	✓	×		GoF	#1
	R691A	×	↓	0	✓↓	×	✓	×		LoF	#2
Brace-I	K938A	✓↑	↓	↑↑	✓↑	✓	✓	×		GoF	#1
	V939A	×	↓	0	×	×	✓	×		LoF	#2
	G943S	×	↓↓	0	×	×	×	✓		LoF	#2
	F945A	× (2)	↓	0	× (2)	×	×	✓		LoF	#2
	D946A	×	↓	0	×	?	×	✓		LoF	#2
Brace-II	Q954A	× (1)	↓	↓	× (1)	✓	✓	×		LoF	#2
	Q954W	×	↓	0	×	×	×	×	✓=	LoF	#2
	L958A	×	↓	0	×	×	✓	×		LoF	#2
	L961A	×	↓	↓↓	✓↑	?	✓	×		LoF	#2

The legend for this table is located on the next page.

In biochemistry assays: mutant does (✓), or does not (×), translocate DNA, slide mono-nucleosomes, or eject nucleosomes from arrays. Activities: =, ↑, ↑↑, ↓, ↓↓: Equal, Moderate or strong, increase or decrease, when compared to WT; 0: No activity; ?: untested.

In genetic experiments: In complementation assays (Genet. Cp), mutant fully (✓), or weakly (✓) complements, or does not complement (×). In dominant-lethality assay (Genet. DL), mutant is dominant lethal (✓), or dominant slow growing (✓), or not impacted (×).

In genomics, ATAC-seq performed (✓), with chromatin accessibility equal (=), moderately (↑) or strongly (↑↑) increased when compared to WT.

Functional classification: GoF = gain-of-function, LoF = loss-of-function, DL = dominant-lethality.

(1): ✓ if in the SAR format, contrasted to: (2): × if in the SAR format.
(*): Experiments documented in Clapier et al., 2016.

Meaning of the shading color, and location in the Results:

	Region #1 alanine substitutions, described in “Alanine Scanning Mutations”
	Region #1 alanine substitutions displaying increased nucleosome ejection, described in “Alanine Scanning Mutations”
	Region #2 alanine substitutions, described in “Alanine Scanning Mutations”
	Region #2 alanine substitutions requiring ARP module restoration, described in “Alanine Scanning Mutations”
	mra mutations, described in “Mutations that Suppress *arp*Δ Mutations”
	Dominant lethal mutations, described in “Mutations that Suppress *arp*Δ Mutations”
	Gain-of-function Cancer-Associated Missense Mutations, described in “Gain-of-function Cancer-Associated Mutations”’
	Loss-of-function Cancer-Associated Missense Mutations, described in “Loss-of-function Cancer-Associated Mutations”’
	Region #1, regulatory region of the SWI/SNF structural hub
	Region #2, implementation region of the SWI/SNF structural hub

Table S2, related to Key Resources Table.

Oligonucleotides used for site-directed mutagenesis and sequencing.

Oligo Name	Oligo Sequence	Source
CC_SDM373PF	GATGAACAGAAAAGAATAGAAAGACCGGCTAAGCAACGTTTAGCTGCTTTG	This paper
CC_SDM373PR	CAAAGCAGCTAAACGTTGCTTAGCCGGTCTTTCTATTCTTTTCTGTTCATC	This paper
CC_SDM382NF	GCAACGTTTAGCTGCTTTGAACTCTAACGATGAGGAGGCTTATTTAAAG	This paper
CC_SDM382NR	CTTTAAATAAGCCTCCTCATCGTTAGAGTTCAAAGCAGCTAAACGTTGC	This paper
CC_SDM382IF2	GCTAAGCAACGTTTAGCTGCTTTGATTTCTAACGATGAGGAGGCTTATTTAAAGC	This paper
CC_SDM382IR2	GCTTTAAATAAGCCTCCTCATCGTTAGAAATCAAAGCAGCTAAACGTTGCTTAGC	This paper
CC_SDM383PF	GCAACGTTTAGCTGCTTTGAAGCCGAACGATGAGGAGGCTTATTTAAAG	This paper
CC_SDM383PR	CTTTAAATAAGCCTCCTCATCGTTCGGCTTCAAAGCAGCTAAACGTTGC	This paper
CC_SDM384KF	GCAACGTTTAGCTGCTTTGAAGTCTAAAGATGAGGAGGCTTATTTAAAG	This paper
CC_SDM384KR	CTTTAAATAAGCCTCCTCATCTTTAGACTTCAAAGCAGCTAAACGTTGC	This paper
CC_SDM384DF	GCAACGTTTAGCTGCTTTGAAGTCTGACGATGAGGAGGCTTATTTAAAG	This paper
CC_SDM384DR	CTTTAAATAAGCCTCCTCATCGTCAGACTTCAAAGCAGCTAAACGTTGC	This paper
CC_SDM385YF	CGTTTAGCTGCTTTGAAGTCTAACTATGAGGAGGCTTATTTAAAGCTTTTAG	This paper
CC_SDM385YR	CTAAAAGCTTTAAATAAGCCTCCTCATAGTTAGACTTCAAAGCAGCTAAACG	This paper
CC_SDM393AF	GATGAGGAGGCTTATTTAAAGCTTGCGGATCAAACTAAGGATACTAG	This paper
CC_SDM393AR	CTAGTATCCTTAGTTTGATCCGCAAGCTTTAAATAAGCCTCCTCATC	This paper
CC_SDM394AF	GAGGAGGCTTATTTAAAGCTTTTAGCGCAAACTAAGGATACTAGAATTAC	This paper
CC_SDM394AR	GTAATTCTAGTATCCTTAGTTTGCGCTAAAAGCTTTAAATAAGCCTCCTC	This paper
CC_SDM397AF	CTTATTTAAAGCTTTTAGATCAAACTGCGGATACTAGAATTACACAGTTATTG	This paper
CC_SDM397AR	CAATAACTGTGTAATTCTAGTATCCGCAGTTTGATCTAAAAGCTTTAAATAAG	This paper
CC_SDMK397delF	GGCTTATTTAAAGCTTTTAGATCAAACTGATACTAGAATTACACAGTTATTGAGGC	This paper
CC_SDMK397delR	GCCTCAATAACTGTGTAATTCTAGTATCAGTTTGATCTAAAAGCTTTAAATAAGCC	This paper
CC_SDM400AF	GCTTTTAGATCAAACTAAGGATACTGCGATTACACAGTTATTGAGGCAGAC	This paper
CC_SDM400AR	GTCTGCCTCAATAACTGTGTAATCGCAGTATCCTTAGTTTGATCTAAAAGC	This paper
CC_SDM400CF	GCTTTTAGATCAAACTAAGGATACTTGCATTACACAGTTATTGAGGCAGAC	This paper
CC_SDM400CR	GTCTGCCTCAATAACTGTGTAATGCAAGTATCCTTAGTTTGATCTAAAAGC	This paper
CC_SDM415AF	GACTAATTCCTTTTTGGATTCTGCGTCGGAGGCTGTTAGAGCACAAC	This paper
CC_SDM415AR	GTTGTGCTCTAACAGCCTCCGACGCAGAATCCAAAAAGGAATTAGTC	This paper
CC_SDM416AF	GACTAATTCCTTTTTGGATTCTTTGGCGGAGGCTGTTAGAGCACAACAAAAC	This paper
CC_SDM416AR	GTTTTGTTGTGCTCTAACAGCCTCCGCCAAAGAATCCAAAAAGGAATTAGTC	This paper
CC_SDMS416MF	GACTAATTCCTTTTTGGATTCTTTGATGGAGGCTGTTAGAGCACAACAAAACG	This paper
CC_SDMS416MR	CGTTTTGTTGTGCTCTAACAGCCTCCATCAAAGAATCCAAAAAGGAATTAGTC	This paper
CC_SDM447AF	GATGAAGAAAGAGAAAAGACTGACGCGTATGAAGTCGCTCATAGGATCAAAG	This paper
CC_SDM447AR	CTTTGATCCTATGAGCGACTTCATACGCGTCAGTCTTTTCTCTTTCTTCATC	This paper
CC_SDM448AF	GATGAAGAAAGAGAAAAGACTGACTATGCGGAAGTCGCTCATAGGATCAAAG	This paper
CC_SDM448AR	CTTTGATCCTATGAGCGACTTCCGCATAGTCAGTCTTTTCTCTTTCTTCATC	This paper
CC_SDM452AF	GACTGACTATTATGAAGTCGCTGCGAGGATCAAAGAAAAAATTGATAAAC	This paper
CC_SDM452AR	GTTTATCAATTTTTTCTTTGATCCTCGCAGCGACTTCATAATAGTCAGTC	This paper
CC_SDMH452YF	GACTGACTATTATGAAGTCGCTTATAGGATCAAAGAAAAAATTGATAAACAACC	This paper
CC_SDMH452YR	GGTTGTTTATCAATTTTTTCTTTGATCCTATAAGCGACTTCATAATAGTCAGTC	This paper
CC_SDM647AF	GCACTGTTGAACTTTGTTTTGCCTGCGATTTTCAATTCTGCGAAGACTTTTG	This paper
CC_SDM647AR	CAAAAGTCTTCGCAGAATTGAAAATCGCAGGCAAAACAAAGTTCAACAGTGC	This paper
CC_SDM655AF	GCCTAAAATTTTCAATTCTGCGAAGACTGCGGAAGATTGGTTTAATACTCC	This paper
CC_SDM655AR	GGAGTATTAAACCAATCTTCCGCAGTCTTCGCAGAATTGAAAATTTTAGGC	This paper
CC_SDM658AF	CAATTCTGCGAAGACTTTTGAAGATGCGTTTAATACTCCATTTGCCAACACCG	This paper
CC_SDM658AR	CGGTGTTGGCAAATGGAGTATTAAACGCATCTTCAAAAGTCTTCGCAGAATTG	This paper
CC_SDM659AF	CTGCGAAGACTTTTGAAGATTGGGCTAATACTCCATTTGCCAACACCGG	This paper
CC_SDM659AR	CCGGTGTTGGCAAATGGAGTATTAGCCCAATCTTCAAAAGTCTTCGCAG	This paper
CC_SDM680MF	CTAGAATTAACGGAAGAAGAAACTATGTTAATTATTAGAAGATTACATAAG	This paper
CC_SDM680MR	CTTATGTAATCTTCTAATAATTAACATAGTTTCTTCTTCCGTTAATTCTAG	This paper
CC_SDM680VF	CTAGAATTAACGGAAGAAGAAACTGTGTTAATTATTAGAAGATTACATAAG	This paper
CC_SDM680VR	CTTATGTAATCTTCTAATAATTAACACAGTTTCTTCTTCCGTTAATTCTAG	This paper
CC_SDM684AF	CGGAAGAAGAAACTCTGTTAATTATTGCGAGATTACATAAGGTGTTAAGGCC	This paper
CC_SDM684AR	GGCCTTAACACCTTATGTAATCTCGCAATAATTAACAGAGTTTCTTCTTCCG	This paper
CC_SDM684WF	CGGAAGAAGAAACTCTGTTAATTATTTGGAGATTACATAAGGTGTTAAGGCC	This paper

CC_SDM684WR	GGCCTTAACACCTTATGTAATCTCCAAATAATTAACAGAGTTTCTTCTTCCG	This paper
CC_SDM685AF	GAAACTCTGTTAATTATTAGAGCGTTACATAAGGTGTTAAGGCCA	This paper
CC_SDM685AR	TGGCCTTAACACCTTATGTAACGCTCTAATAATTAACAGAGTTTC	This paper
CC_SDM685HF	GAAACTCTGTTAATTATTAGACATTTACATAAGGTGTTAAGGCCA	This paper
CC_SDM685HR	TGGCCTTAACACCTTATGTAAATGTCTAATAATTAACAGAGTTTC	This paper
CC_SDM687AF	GAAACTCTGTTAATTATTAGAAGATTAGCGAAGGTGTTAAGGCCATTTTTA	This paper
CC_SDM687AR	TAAAAATGGCCTTAACACCTTCGCTAATCTTCTAATAATTAACAGAGTTTC	This paper
CC_SDM687YF2	CTCTGTTAATTATTAGAAGATTATATAAGGTGTTAAGGCCATTTTTATTGCGTCG	This paper
CC_SDM687YR2	CGACGCAATAAAAATGGCCTTAACACCTTATATAATCTTCTAATAATTAACAGAG	This paper
CC_SDM688AF	CTCTGTTAATTATTAGAAGATTACATGCGGTGTTAAGGCCATTTTTATTGCG	This paper
CC_SDM688AR	CGCAATAAAAATGGCCTTAACACCGCATGTAATCTTCTAATAATTAACAGAG	This paper
CC_SDM688TF	CTGTTAATTATTAGAAGATTACATACCGTGTTAAGGCCATTTTTATTGCG	This paper
CC_SDM688TR	CGCAATAAAAATGGCCTTAACACGGTATGTAATCTTCTAATAATTAACAG	This paper
CC_SDM691AF	TTATTAGAAGATTACATAAGGTGTTAGCGCCATTTTTATTGCGTCGTTTG	This paper
CC_SDM691AR	CAAACGACGCAATAAAAATGGCGCTAACACCTTATGTAATCTTCTAATAA	This paper
CC_SDM938AF	GCAGAAATTGGACATTGACGGCGCGGTTATCCAGGCAGGTAAGTTTGATAAC	This paper
CC_SDM938AR	GTTATCAAACTTACCTGCCTGGATAACCGCGCCGTCAATGTCCAATTTCTGC	This paper
CC_SDM939AF	GAAATTGGACATTGACGGCAAGGCGATCCAGGCAGGTAAGTTTGATAACAAATC	This paper
CC_SDM939AR	GATTTGTTATCAAACTTACCTGCCTGGATCGCCTTGCCGTCAATGTCCAATTTC	This paper
CC_SDM943SF	CATTGACGGCAAGGTTATCCAGGCAAGCAAGTTTGATAACAAATCAACTGCAG	This paper
CC_SDM943SR	CTGCAGTTGATTTGTTATCAAACTTGCTTGCCTGGATAACCTTGCCGTCAATG	This paper
CC_SDM945AF	GGCAAGGTTATCCAGGCAGGTAAGGCGGATAACAAATCAACTGCAGAAGAG	This paper
CC_SDM945AR	CTCTTCTGCAGTTGATTTGTTATCCGCCTTACCTGCCTGGATAACCTTGCC	This paper
CC_SDM946AF	CAAGGTTATCCAGGCAGGTAAGTTTGCGAACAAATCAACTGCAGAAGAGCAAG	This paper
CC_SDM946AR	CTTGCTCTTCTGCAGTTGATTTGTTCGCAAACTTACCTGCCTGGATAACCTTG	This paper
CC_SDM954AF	GATAACAAATCAACTGCAGAAGAGGCGGAAGCTTTTTTGAGAAGATTG	This paper
CC_SDM954AR	CAATCTTCTCAAAAAAGCTTCCGCCTCTTCTGCAGTTGATTTGTTATC	This paper
CC_SDM954WF	GATAACAAATCAACTGCAGAAGAGTGGGAAGCTTTTTTGAGAAGATTG	This paper
CC_SDM954WR	CAATCTTCTCAAAAAAGCTTCCCACTCTTCTGCAGTTGATTTGTTATC	This paper
CC_SDM958AF	GCAGAAGAGCAAGAAGCTTTTGCGAGAAGATTGATCGAAAGTGAAACG	This paper
CC_SDM958AR	CGTTTCACTTTCGATCAATCTTCTCGCAAAAGCTTCTTGCTCTTCTGC	This paper
CC_SDM961AF	GAGCAAGAAGCTTTTTTGAGAAGAGCGATCGAAAGTGAAACGAACAGG	This paper
CC_SDM961AR	CCTGTTCGTTTCACTTTCGATCGCTCTTCTCAAAAAAGCTTCTTGCTC	This paper
CC_TR3Fseq	CATACTGCCGCCCTGACCGAC	This paper
CC_STH1R03	GTAATTCAAACTTGCCCGCTACTCTG	This paper
CC_STH1BsFseq	CTAGAGCCGGTGGTTTGGGTTTG	This paper

Note: L392V, L392P, ΔDL, E676Q, E677G, L681F, and L681S constructs were derived from plasmids used in previous published (Clapier et al., 2016) and unpublished studies, and will be provided upon request.
